# Advancement and obstacles in microfluidics-based isolation of extracellular vesicles

**DOI:** 10.1007/s00216-022-04362-3

**Published:** 2022-10-26

**Authors:** Megan Havers, Axel Broman, Andreas Lenshof, Thomas Laurell

**Affiliations:** Biomedical Engineering Department, Lund University, Ole Römers väg 3, 221 00 Lund, Sweden

**Keywords:** Extracellular vesicles, Nanoparticle isolation, Microfluidics

## Abstract

There is a great need for techniques which enable reproducible separation of extracellular vesicles (EVs) from biofluids with high recovery, purity and throughput. The development of new techniques for isolation of EVs from minute sample volumes is instrumental in enabling EV-based biomarker profiling in large biobank cohorts and paves the way to improved diagnostic profiles in precision medicine. Recent advances in microfluidics-based devices offer a toolbox for separating EVs from small sample volumes. Microfluidic devices that have been used in EV isolation utilise different fundamental principles and rely largely on benefits of scaling laws as the biofluid processing is miniaturised to chip level. Here, we review the progress in the practicality and performance of both passive devices (such as mechanical filtering and hydrodynamic focusing) and active devices (using magnetic, electric or acoustic fields). As it stands, many microfluidic devices isolate intact EV populations at higher purities than centrifugation, precipitation or size-exclusion chromatography. However, this comes at a cost. We address challenges (in particular low throughput, clogging risks and ability to process biofluids) and highlight the need for more improvements in microfluidic devices. Finally, we conclude that there is a need to refine and standardise these lab-on-a-chip techniques to meet the growing interest in the diagnostic and therapeutic value of purified EVs.

**Graphical abstract:**

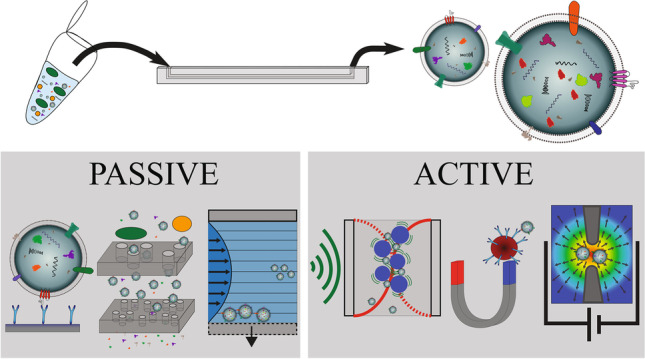

## Introduction

**Fig. 1:**
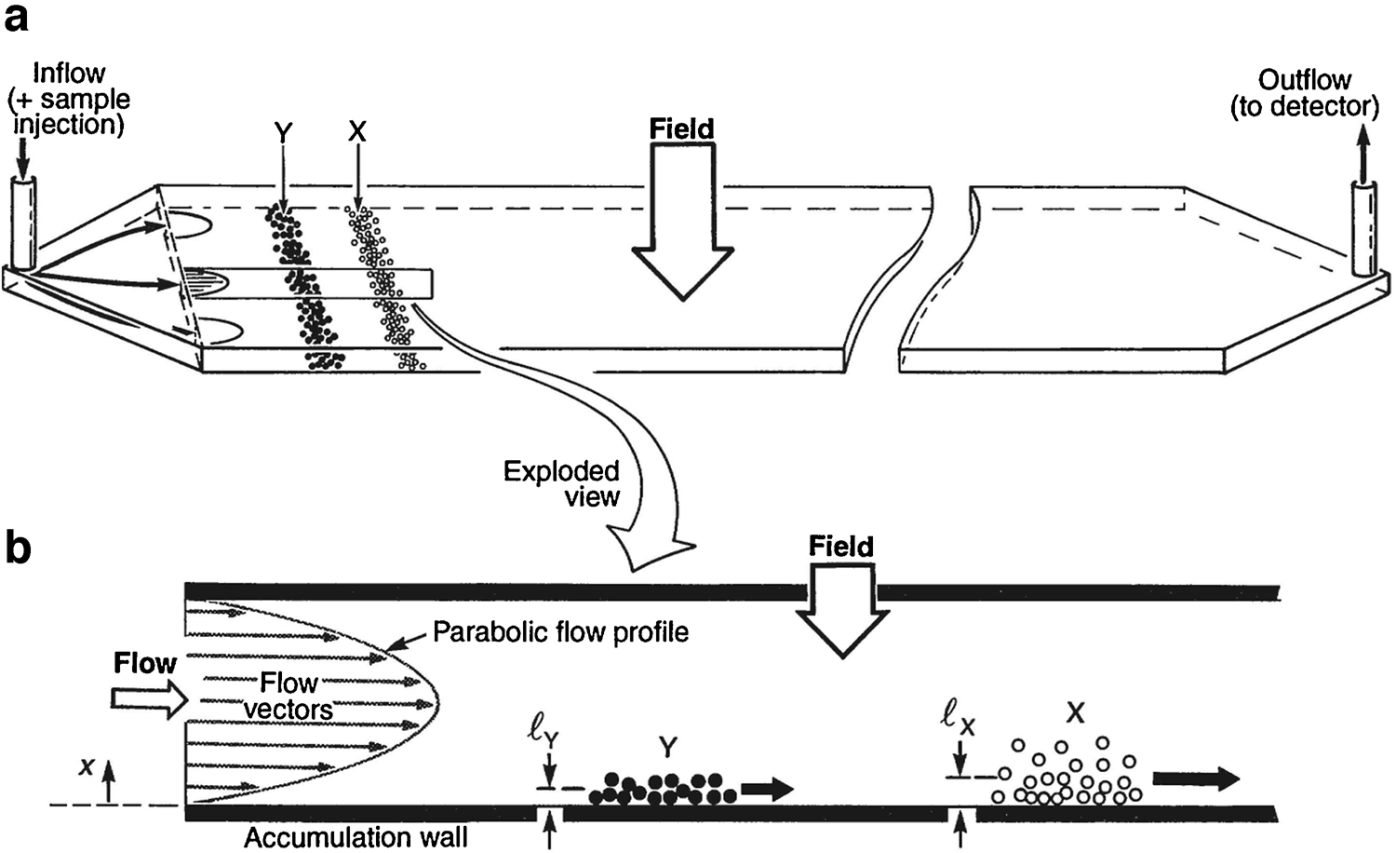
Schematic of FFF of two component particle populations X and Y via the normal mode operation with view **a** the microfluidic channel with mixed inflow and sorted outflow and **b** the flow profile across the channel and the different wall displacement of two components due to different field interactions. Illustration from [2]. Reprinted with permission from AAAS

The term microfluidic devices covers an excitingly broad range of fluidic concepts and applications. On a basic level, they describe systems which handle fluids in microlitre volumes within structures of micrometre-scale dimensions. The power of these kinds of devices, to perform high-performance separations of particle populations in a fluid, was demonstrated with field-flow fractionation (FFF) pioneered by Giddings [1]. In FFF (see Fig. 1), deterministic fluid handling controls particle motion along flow vectors with only diffusion (driven by Brownian motion) affecting their mean distance above the accumulation wall with respect to the field. The elution time of populations of particles with different Brownian motion will vary and allow separation of a wide range of colloidal sizes and materials. FFF has the potential to use different fields (including but not limited to sedimentation, magnetic, dielectric, acoustic, cross-flow, shear and concentration gradients), which leaves many parameters to be optimised and devices still unexplored [1].

Recent advances in fabrication and modes of operation have driven increasing interest in applications for biological nanoparticle processing. This review focuses on microfluidic techniques for isolating extracellular vesicles, an area of active research which lacks standards for performance characterisation. EV separation from biofluids requires short processing times and low sample volumes with the added challenges of recovery and specificity [3]. However, microfluidic techniques show great promise in tackling these challenges. In general, microfluidic devices, as with FFF, rely on microscale deterministic fluidic handling; however, they can be described as ‘passive’ or ‘active’, depending on the separation mechanism. Antibodies can be used to manipulate biological species, whereas label-free techniques depend on physical properties. In this way, we can categorise microfluidic devices, comparing their versatility and highlighting areas which show the most promise for EV separation.

## Extracellular vesicle isolation

**Fig. 2:**
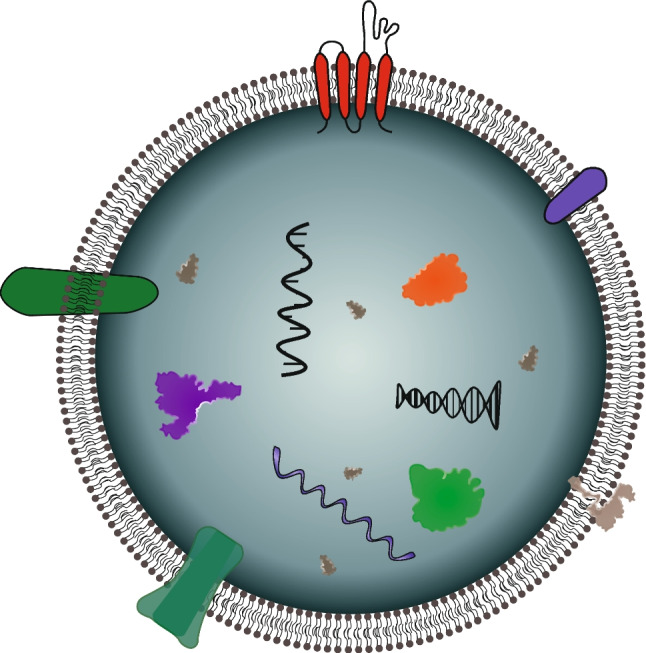
Schematic cross section of an extracellular vesicle. The lipid bilayer membrane is represented containing various proteins (represented by colourful irregular shapes in the membrane or vesicle), RNA and DNAs (represented by nucleic acid helixes inside the vesicle), inherited from the cell of origin

Extracellular vesicles (EVs) are particles possessing a lipid bilayer membrane and originating from cells, but which do not themselves have a functional nucleus, as illustrated in Fig. 2 [3]. Evidence suggests that such vesicles hold the key to information transport between cells, but knowledge of their contents is still evolving [3]. Historically, EVs have been subpopulated by their cellular mechanism of origins into three groups with varying sizes. Exosomes (30–100 nm) and microvesicles (100–1000 nm) are released constitutively or by activation. Apoptotic bodies (50–2000 nm) are generated during cell death [4]. Since EVs are studied from cell cultures and biological fluids, where biogenesis cannot be directly observed, it is impractical to subpopulate in this way. It is more useful to categorise EVs by size, density, biochemical composition, or descriptions of conditions or the cell of origin [3]. Biochemical composition can include the expression of cell surface markers on the EVs, like tetraspanins CD63 and/or CD81. Furthermore, EVs may contain proteins, RNAs, DNA and other biomarkers reflecting a patient’s health status.

## Standard EV isolation methods

The diagnostic potential of EVs is already considered a Holy Grail and is being explored extensively in the search for disease-related biomarkers. Current methods of isolating EVs have significant limitations in quantification from small volumes of biofluids and co-isolated proteins impede diagnostic capabilities. The methods discussed in this section are the current gold standards, yet it is evident that these will need to be complemented by a new generation of reliable and versatile devices like those detailed in the main body of this report.

### Ultracentrifugation

**Fig. 3:**
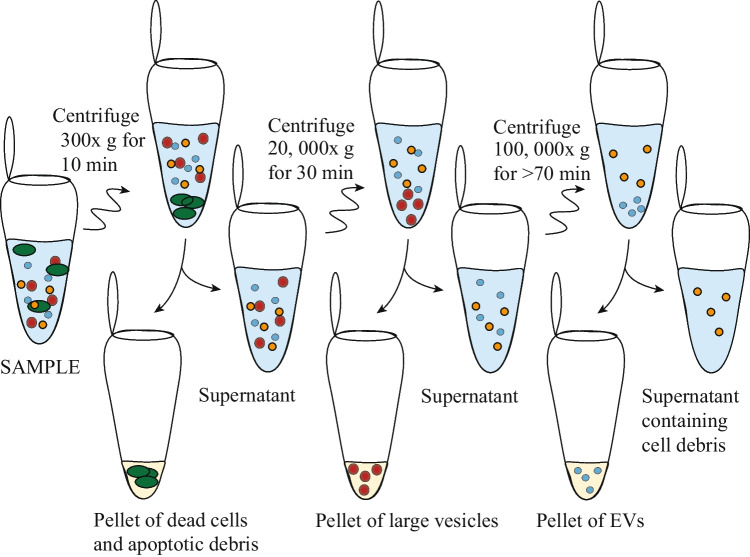
Ultracentrifugation protocol for isolating EVs from body fluid or conditioned medium, taking several hours. Illustration inspired by [4]

Much of the progress in EV research has relied on ultracentrifugation (UC) to purify and isolate them from biofluids. As in traditional centrifugation for cell handling, biological species can be separated by their density and hydrodynamic size. However, since EVs are very small, extremely high centrifugal forces (100,000–120,000 × g) over extended time periods are required to pellet EVs, and successive UC steps (as shown in Fig. 3) are required to obtain sub-populations [5]. Following UC, EVs may be further purified by density gradient floatation (DGF), which removes non-membranous particles using a gradient of density solution. However, the high sucrose concentrations can damage EV integrity [5].

Ultracentrifugation is a flexible technique and is available in most medical research labs. However, adding UC steps adds hours to the processing time, is labour intensive, requires relatively large sample volumes, has modest and variable recovery and is far from point of care which limits its practical use in diagnostics. Additionally, high-speed centrifugation may induce EV coalescence, leading to erroneous conclusions regarding EV size, concentration and phenotype [6].

### Precipitation

To overcome the time limitation of ultracentrifugation, many kinds of precipitation methods have been developed. These involve additives which speed up the pelleting of EVs such as polymers [4], beads and antibodies. There are many commercialised kits including the Urine Exosome RNA Isolation Kit (NORGEN Biotek Corp., Thorold, ON, Canada), Total Exosome Isolation Solution (ThermoFisher, Waltham, MA, USA), Exoquick-TC (System Biosciences, Palo Alto, CA, USA) and RIBO™ Exosome Isolation Reagent (RIBO Guangzhou, China). These tend to require a several-hour incubation followed by a low-speed ultracentrifugation, so an advantage is that they are not particularly labour intensive [5]. Although these methods have medium–high recovery, the purity may be reduced by additives [3] and co-isolated lipoproteins [5]. Comparative studies have found variable performance between kits and depending on the biofluid [7, 8], and the International Society for Extracellular Vesicles has cautioned against their use [3].

### Size-exclusion chromatography

Size-exclusion chromatography (SEC) has been cited as the gold standard of the last decade [9]. SEC has some significant advantages over ultracentrifugation, such as processing smaller volumes with fewer manual handling errors. Relying on chromatography principles, different species exhibit predictably different elution times from a column, which is biassed towards certain physical and biochemical properties. SEC columns have been designed to have EVs of particular size ranges coming out into discreet fractions. In qEV products (IZON Science LTD, New Zealand), the user can choose a column which is biassed towards 35-nm or 70-nm EVs.

The SEC columns can take under 15 min to isolate EVs with intermediate recovery and specificity [3]. Consistent, standardisable results can make it preferable to UC techniques. SEC is also preferable to precipitation techniques due to lower abundance of contaminating proteins [9]. However, the devices are bulky and expensive with disposable columns. The sample volume must suit the column and you need to know what size EVs you are aiming for, so SEC is sub-optimal for many applications and risks skewed results. EVs risk getting stuck to the low-molecular-weight filters in the device, and if a large force is applied, it could damage larger vesicles or platelets [5].

## Microfluidic isolation techniques

With escalating biomedical EV research, there is a need for separation methods which are reliable, fast, affordable, automated, high yield and high purity and can collect specific subsets of vesicles. Microfluidics is a field of engineering which typically handles fluid volumes in the order of microlitres. There are many reasons why scaling down in this way is advantageous for isolating small particles. Firstly, it creates the opportunity to perform diagnostics with scarce biofluids from animals, patients and cell cultures. Secondly, the deterministic fluid handling in microfluidic systems allows for superior control of the sample and intrinsic reproducibility. The separation of particles in microfluidic systems can be predicted due to the laminar flow profiles where a difference in size results in different displacement.

Ideally, a standardised microfluidic system will be developed to handle any biological sample, no matter the volume, density or concentration of both vesicles and soluble proteins. Although microfluidic devices still have obstacles to overcome, they have advanced the field of EV isolation from smaller sample volumes and a range of biofluids. In the following discussion, we consider devices as ‘passive’ or ‘active’ if external forces are required and we discuss the diverse advantages and limitations within these groups. Comparing the devices is difficult since purity, throughput, recovery and the analytical techniques used to quantify performance vary. Nevertheless, this report aims to advise on the usefulness of techniques in different applications and inspire the development of the next generation of microfluidic EV isolation methods.

### Immunoaffinity-based techniques

In microfluidic isolation of EVs, antibodies may be used as a tool to adhere an EV with a complementary target to a surface or particle (for example magnetic or polystyrene beads). CD63, CD9 and CD81 are tetraspanins widely accepted as EV biomarkers, which allow immuno-selective isolation and enrichment of EVs. EVs can also be targeted based on markers from their parent cell for more specific isolation dependent on the cellular origin. Markers that are commonly targeted in such a way include epidermal growth factor receptor (EGFR) and epithelial cellular adhesion molecule (EpCAM) [10].

**Fig. 4:**
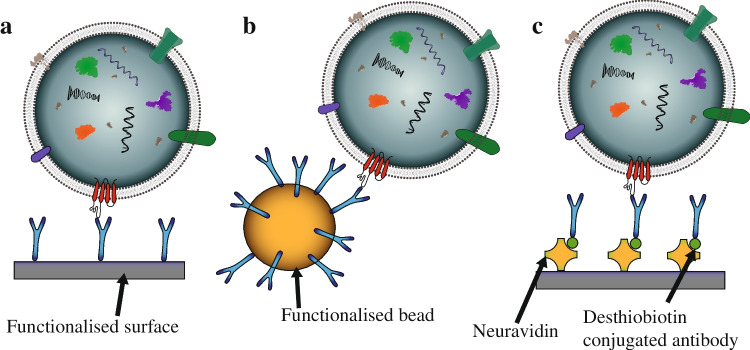
Schematic of three different iterations of immunoaffinity-based isolation devices, where a targeted EV is assumed to possess the surface marker for the complementary antibody. **a** A functionalised surface, **b** a functionalised bead and **c** an example of more complex functionalisation to also allow EV release

Immunoaffinity capture (IC) in microfluidics-based devices [10–14] falls into two categories: functionalised surfaces or functionalised beads in a suspension. Figure 4 summarises common configurations used in EV isolation. Figure 4a illustrates how an EV can be retained at a surface when that surface has been functionalised with appropriate antibodies for the target EV biomarkers, as demonstrated in 2010 by Chen et al. [11]. Similarly, the surface of a particle can be functionalised, as illustrated in Fig. 4b, then manipulated after capture to recover EVs [13]. Early approaches relied on biotin-avidin conjugation, which makes the binding irreversible under physiological conditions. However, Lo et al. demonstrated that desthiobiotin has a lower binding affinity than biotin and can be used in EV isolation, after which it can be replaced by biotin in a competitive binding step to release the EVs [10]; see Fig. 4c.

Following isolation and enrichment of EVs, antibodies can be utilised for analysis by ELISAs, fluorescence or lysing prior to analysis of proteins and nucleic acids. The potential for on-chip quantification makes these techniques flexible and highly desirable for diagnostics [15]. However, the highly selective isolation of EVs has the downside that EVs without a target marker are lost and so recovery is variable. There is also a risk of erroneous conclusions from analysis unless studies include IgG controls and to account for non-specific protein/lipid corona [16]. Since EV concentration and biomarkers can vary hugely, IC methods should be complemented with those allowing separation by physical properties like size and charge, as addressed by the other techniques.

### Passive devices

The following section will highlight passive devices for extracellular vesicle separation, where separation can be achieved by smart design to influence microfluidic/nanoparticle interactions. Such devices only require flow control and no external force fields or user interfaces, making them attractive in point-of-care applications. However, many of these require slow flow rates and/or complex fabrication.

#### Mechanical filtering with micro- and nanostructures

Traditionally, filtering uses a porous mechanical barrier to separate larger particles from smaller ones in a fluid. Using micro- or nano-scale devices, vesicle-sized particles can be separated. Clogging is a major risk with filters, especially for biological samples which are adhesive and contain particles which vary in size by several orders or magnitudes. The risk can be mitigated by designs letting large particles through or using sequential filters.

**Fig. 5:**
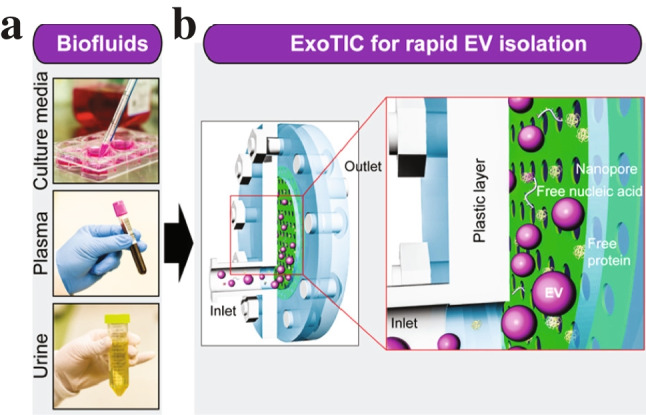
The ExoTIC device. **a** EVs can be isolated from various biofluids. **b** The EV suspensions are passed through a nanoporous membrane. Free protein and nucleic acids can pass through the membrane, whilst EVs cannot. Adapted with permission from [17]. Copyright 2017 American Chemical Society

A common way of realising a microfluidic EV filter is to use a series of membranes with nanometre-sized pores, known as nanoporous membranes, with different pore sizes to fractionate particles by size. An example is the exosome total isolation chip (ExoTIC) [17], which is a device that separates small EVs (see Fig. 5). A biofluid is prefiltered (< 220 nm) to remove larger debris before it is run through the nanoporous membrane (30–200 nm), thus enriching EVs via sequential filtering. Downstream analysis can be done after EVs are washed off the membrane. A 5–10-mL sample was processed under 3 h, with 90% capture efficiency of small EVs. Liang et al. [18] demonstrated the integration of a nanoporous membrane device with on-chip ELISA to confirm isolation of exosomes with CD63.

**Fig. 6:**
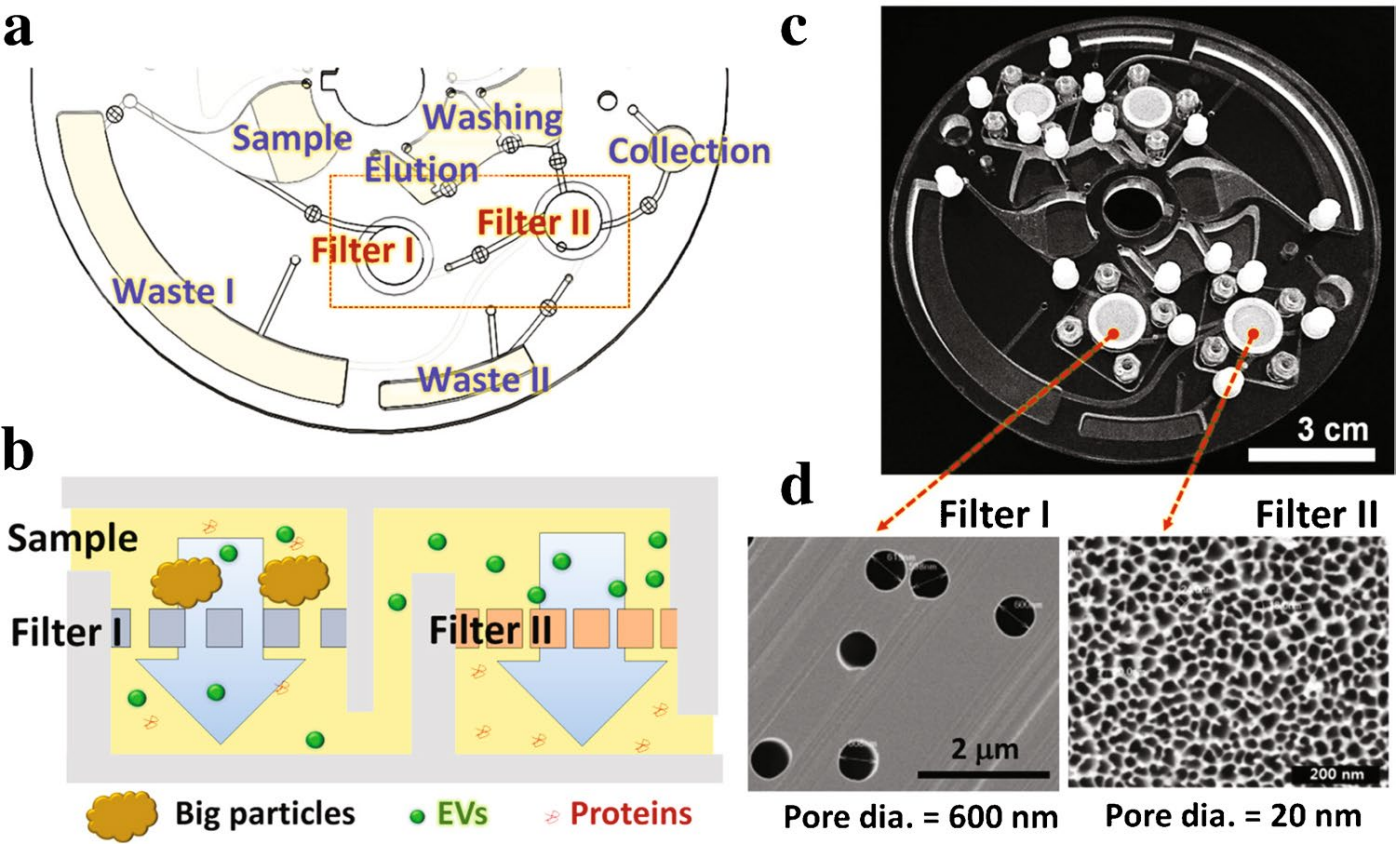
The ExoDisc device. **a** Schematic of the spinning disc with sample, waste and collection chambers as well as the two filters shown in (**b**). Sequential filtering of EVs from big particles and proteins. **c** Image of the device, which has two identical units allowing parallel processing. **d** SEM images of the nanoporous membranes in each filter. Adapted with permission from [19]. Copyright 2017 American Chemical Society

EV separation with nanoporous membranes can be sped up with centrifugal forces in the ExoDisc [19]; see Fig. 6. A double filtration mechanism, along with wash and waste chambers, is fitted inside a spinning disc, which controls the fluid flow. The ExoDisc was able to process a sample in 30 min for exosome isolation, and 1 h if combined with on-chip ELISA. EVs are only exposed to a centrifugal force of 500 × g, much lower than in UC.

**Fig. 7:**
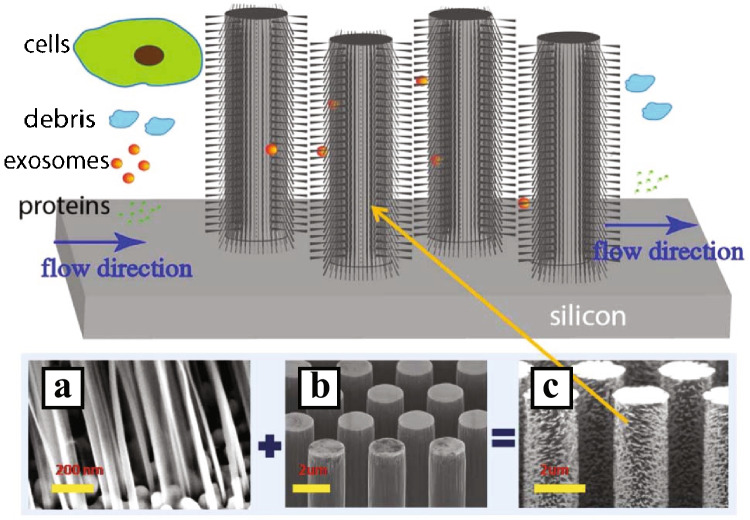
Ciliated micropillars for EV isolation. The spacing of the pillars filters out larger particles. The spacing of the cilia allows for capture of EVs, whilst letting free protein through. **a**–**c** SEM images of the micropillars. Reproduced from [20] with permission from the Royal Society of Chemistry

Filters that probe particles in the size range of EVs can also be made using patterns of micro- and nanostructures. Wang et al. [20] created a pattern of ciliated micropillars that functions as a filter (see Fig. 7) where exosome-sized particles are small enough to enter between the pillars into the finer mesh created by the cilia. Proteins in the solution are washed away as they are too small to be caught in the cilia, whilst the pillar spacing blocks cells and debris from entering between the pillars.

**Fig. 8:**
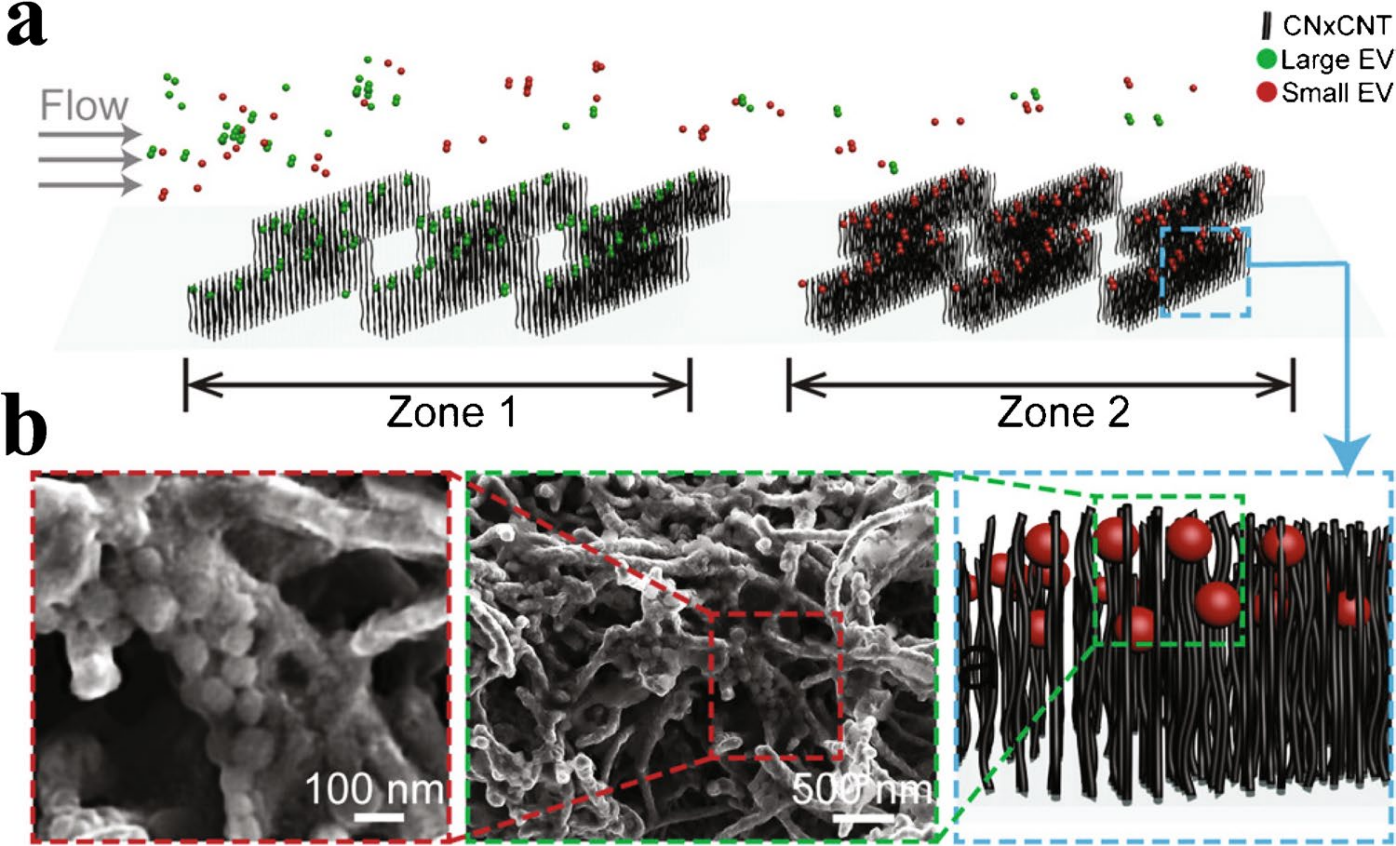
**a** Schematic of patches of carbon nanotubes for capture of EVs. **b** SEM images of the arrays, where the intertubular distance allows for capture of differently sized EVs. Reprinted with permission from [21]. Copyright 2020 American Chemical Society

Yeh et al. [21] printed patches of carbon nanotubes with matching spacing; see Fig. 8. The herringbone structure enhances mixing, and it was possible to run at flow rates of 5–1000 μL/min with capture efficiencies ranging from 10 to 55%. However, the EVs were not eluted from the structures, but instead, cells were grown on top of the patches to study cellular EV uptake. This makes the device unsuitable for isolation of EVs for other downstream analysis methods.

#### Functionalised surfaces

There are many examples of simple microfluidic devices which have incorporated antibodies as functionalised surface coatings to allow selective isolation of EVs; whilst functionalisation requires antibodies and time, only small volumes need to be used. In passive devices using antibodies, microfluidics can be designed to enhance mixing and allow automated washing not possible with traditional techniques.

The pioneering work of Chen et al. [11] demonstrated the capture of EVs from serum using an anti-CD63 functionalised microchannel. The device handled 10–400 μL of sample at 16 μL/min; however, the functionalisation step took more than 2 h [11]. The EVs were then either visualised by SEM or lysed for RNA analysis, with 42–92% isolation efficiency.

The sensitivity of IC surface devices can be improved with nanostructures. Increasing surface area increases the chance of EV interaction and isolation. Zhang et al. [14] demonstrated a limit of detection of 50 exosomes/mL by integrating a graphene oxide/polydopamine nano-interface, with low non-specific protein absorption.

**Fig. 9:**
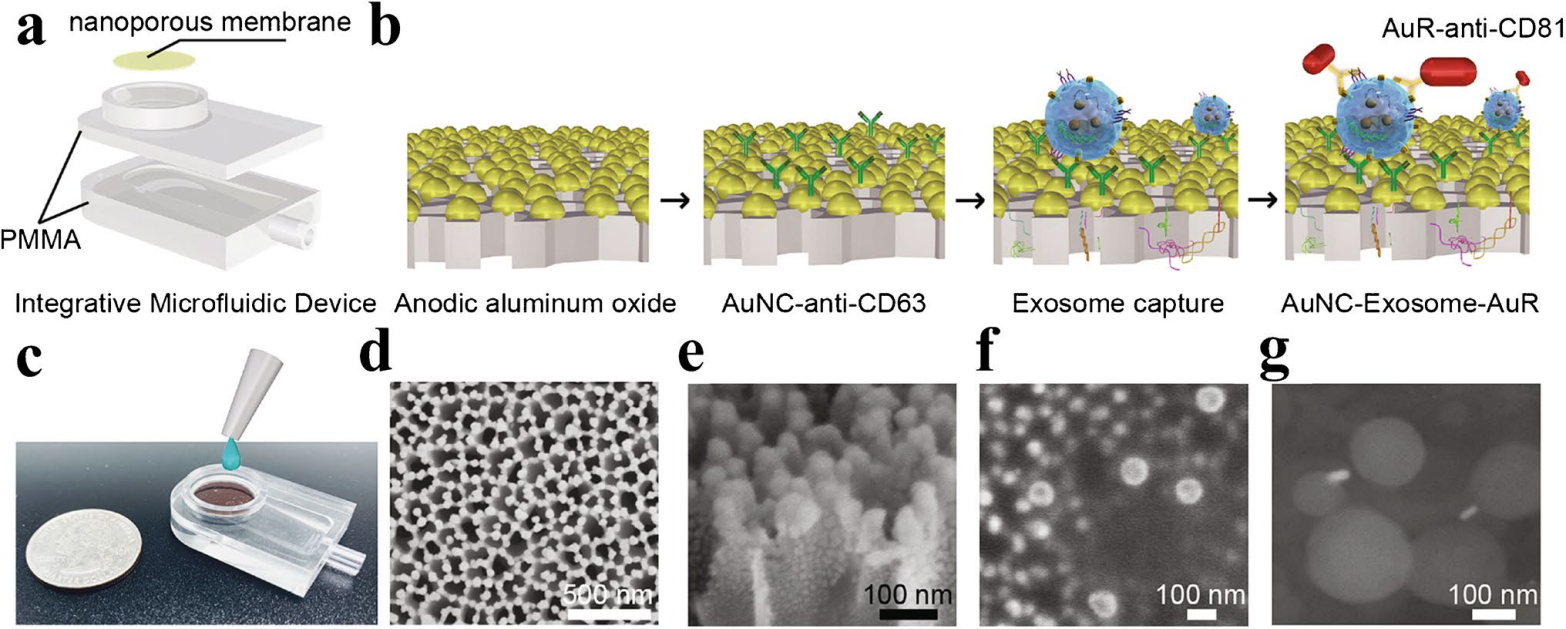
Functionalised nanoporous membrane for specific isolation and detection of exosomes. **a** Design of the device. **b** Schematic illustration of the steps in the in-situ detection of an exosome. **c** Image of the integrative microfluidic device.** d** SEM image of Au nanoparticles deposited on anodic aluminium oxide membrane with a thickness of 50 nm, with **e** the side view of Au coating. **f **SEM image of the captured exosomes on the membrane, and **g** SEM image of the formed complex containing exosomes bound to Au nanoclusters and nanorods. Reprinted from [22], Copyright 2013, with permission from Elsevier

Yang et al. [22] demonstrated a nanoporous membrane coated with gold nanoparticles conjugated with anti-CD63 antibodies; see Fig. 9. The device was able to process 5 mL of urine in 30 min, yielding around 5 × 10^9^ particles. Another filtration device combined with aptamers that bind to CD63 was developed by Dong et al. [23]. This device has the benefit of being able to work with small sample volumes and a low limit of detection (8.9 × 10^3^ EVs per mL). Combined with fluorescence detection of EVs, the processing time of 20 μL of sample was 2 h.

**Fig. 10:**
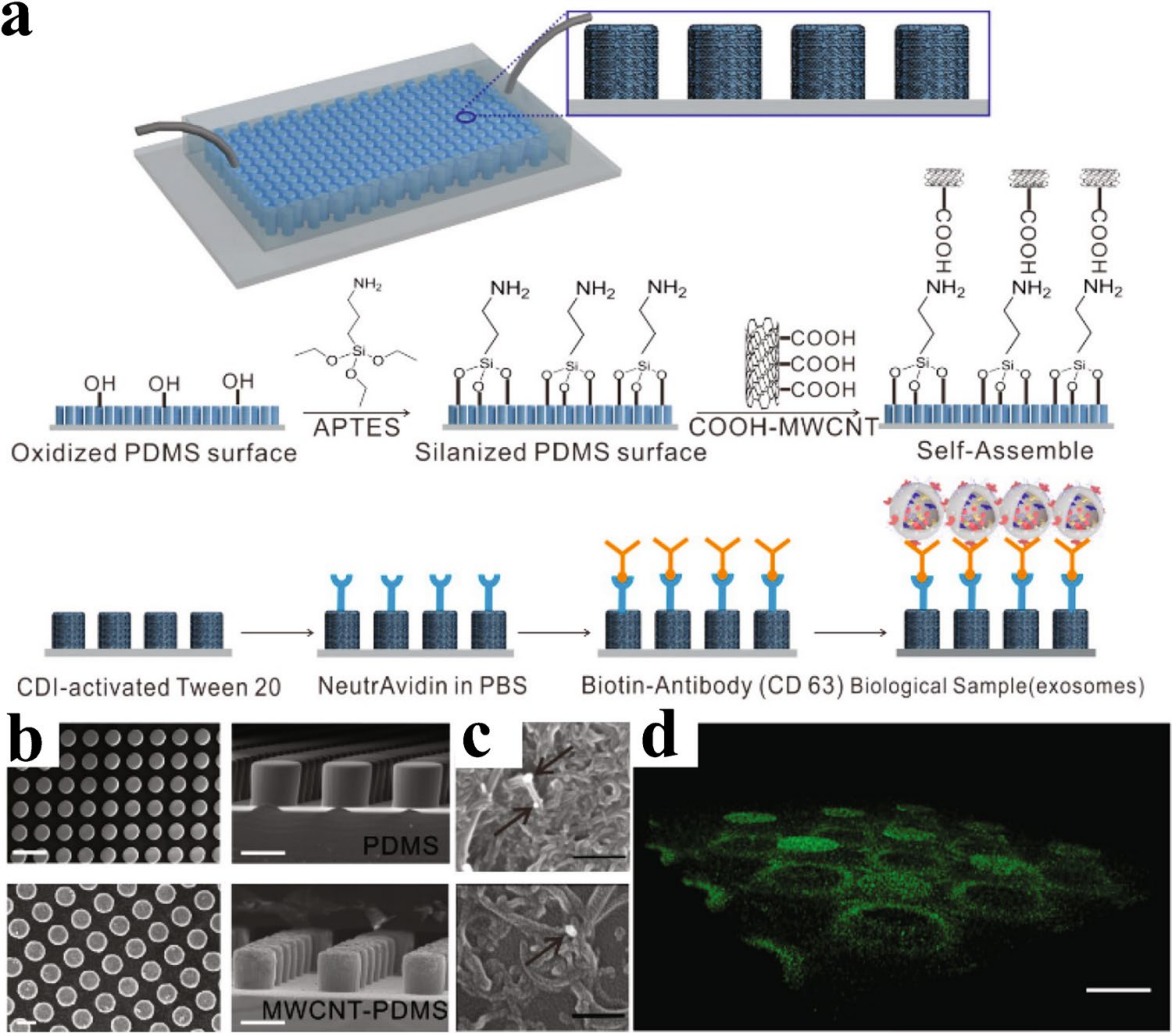
Array of functionalised PDMS micropillars. **a** Schematic of the micropillar array and the process for functionalisation of the pillars. **b** SEM images of the device. **c** SEM images of captured EVs. **d** Fluorescent three-dimensional image of the device stained with avidin-FITC. Scale bar, 50 μm. Reprinted with permission from [24]. Copyright 2017 American Chemical Society

**Fig. 11:**
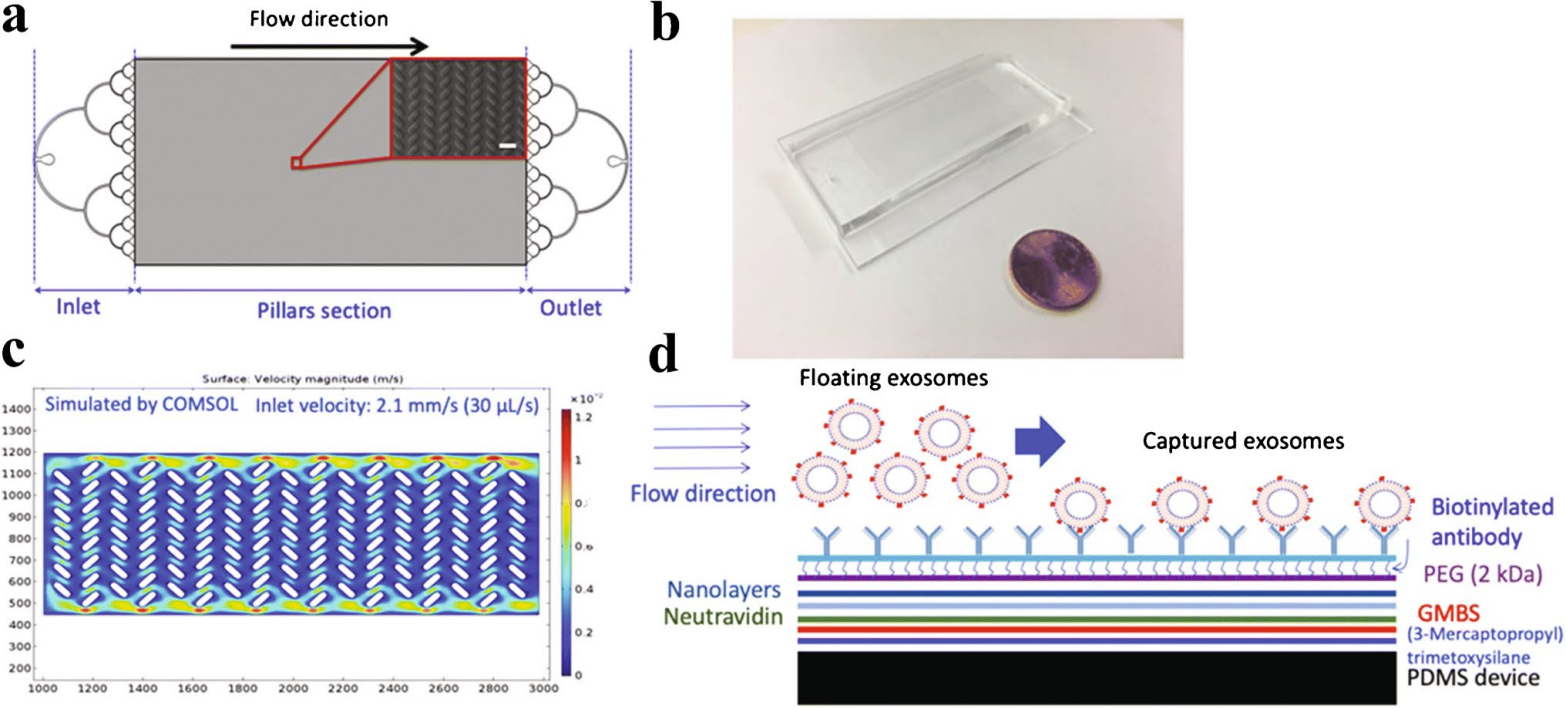
Functionalised micropillars in a zigzag pattern for enhanced interaction between the pillars and the EVs. **a** Schematic of the device, including a SEM image of the pillars. The device contains approximately 100,000 pillars. **b** Picture of the device, with a penny for size comparison. **c** Simulation of the flow in the device. **d** Schematic of the coating layers. Reprinted by permission from [25], Springer Nature, *Biomedical Microdevices*, Copyright 2020

Wang et al. [24] demonstrated isolation of exosomes using an array of functionalised PDMS micropillars; see Fig. 10. Later, Kamyabi et al. [25] used coated pillars for tumour-derived EV isolation and subsequent DNA analysis, utilising a zigzag pattern to increase the interaction between the pillars and the EVs; see Fig. 11. They were able to process 2 mL of plasma in 1.5 h, yielding 2–14 ng of DNA.

For devices with optimised specificity, the detection can be incorporated into the device. ExoChip is an IC device developed with on-chip fluorescence imaging, which uses expanding and contracting microchannels to enhance mixing rather than the herringbone structure used by Chen et al. Using the ExoChip, EVs in a 400-μL sample were captured at 8 μL/min and enabled the detection of 15–18 μg of total proteins and 10–15 ng of nucleic acids [12].

More recently, specialised sequential reagent steps have been utilised to allow triggered release of EVs from the surface, which makes downstream analysis possible. This device could process 1.2 mL of plasma or 10 mL of cell culture medium in an hour. The capture efficiency decreased for flow rates above 10 mL/h, and a lower throughput for higher viscosity medium was observed, due to reduced binding of antibody to antigens under high shear stress. This device is one of the few IC techniques which allows further study of intact EVs via nanoparticle tracking analysis (NTA) as well as studying the uptake of isolated EVs in cancer cells [10].

In summary, IC-based approaches can offer specificity in EV isolation and increase purity. However, it adds complexity to device fabrication and will only isolate EVs that express the targeted surface marker, which may bias downstream analysis.

#### Hydrodynamic focusing

**Fig. 12:**
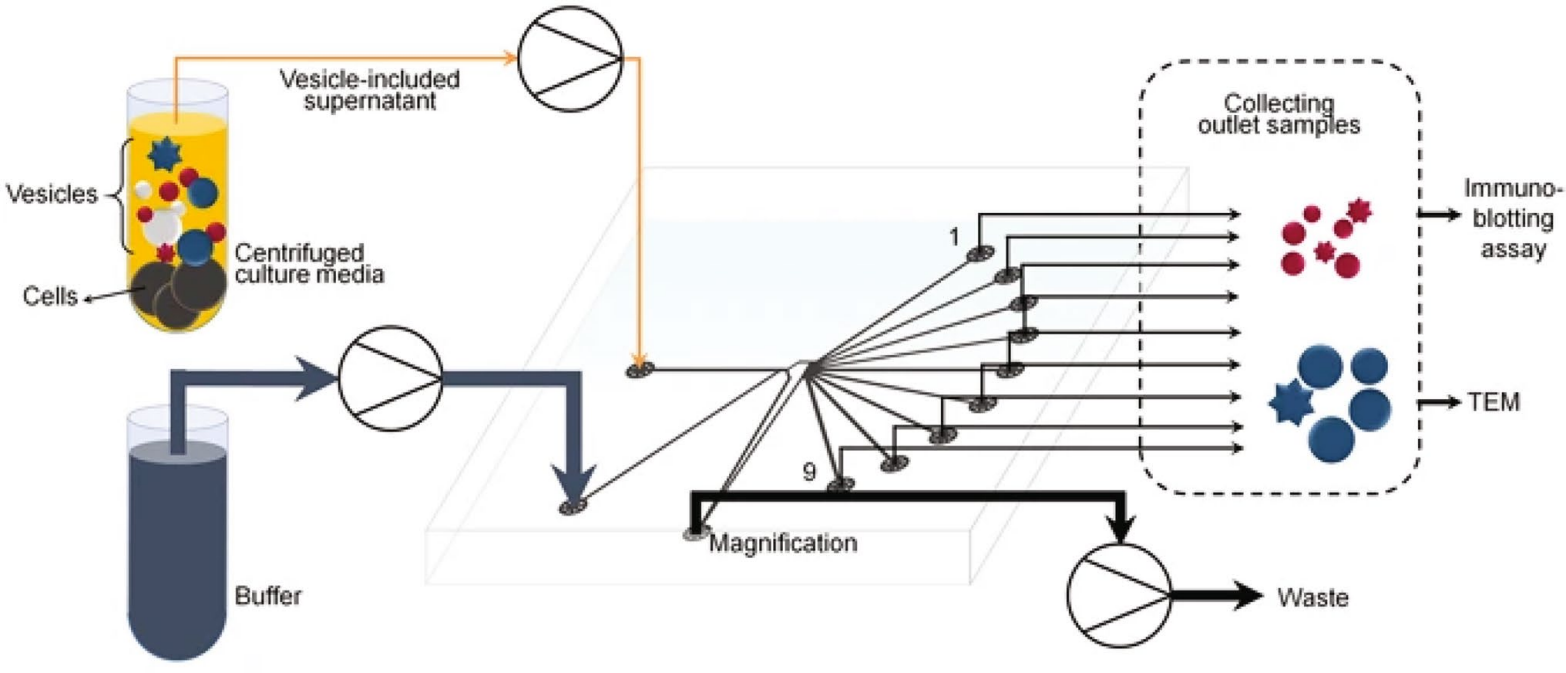
Schematic of pinched-flow fractionation. Adapted with permission from [26], Copyright 2017, the authors

Hydrodynamic focusing is arguably the simplest class of microfluidic separation techniques. Without external fields or extensive fabrication, deterministic fluid flow is used to separate particles by size. Pinch-flow fractionation with a magnification flow channel (see Fig. 12) has separated apoptotic bodies from EVs in cell media, in 25 min (at 200 μL/min). However, all particles below 200 nm were co-isolated [26].

**Fig. 13:**
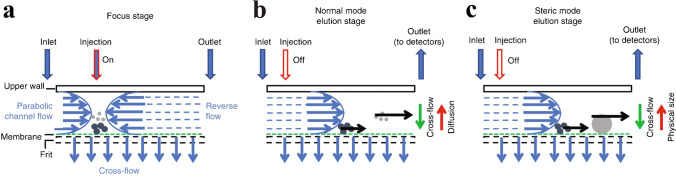
Schematic of AsFlFFF stages. **a** The sample is injected and focused between the two opposing flows (parabolic channel flow and reverse flow) in the channel. In the elution stages, the parabolic channel from inlet to outlet caused the sequential elution of particles of different sizes. **b** The particles reach heights depending on their diffusion coefficient (normal mode). **c** For larger particles, diffusion is negligible and their position in the channel depends on physical size (steric mode). Reprinted by permission from [27], Springer Nature, *Nature Protocols*, Copyright 2019

Asymmetric-flow field-flow fractionation (sometimes called AF4 or AsFlFFF) combines cross-flow with parabolic channel flow, as shown in Fig. 13. Particles are separated by size due to either their diffusion coefficient (normal mode) or their physical size (steric mode). This technique is rapid, highly reproducible and can mimic physiological conditions, unlike density gradient flotation (DGF) and other manual techniques [27].

**Fig. 14:**
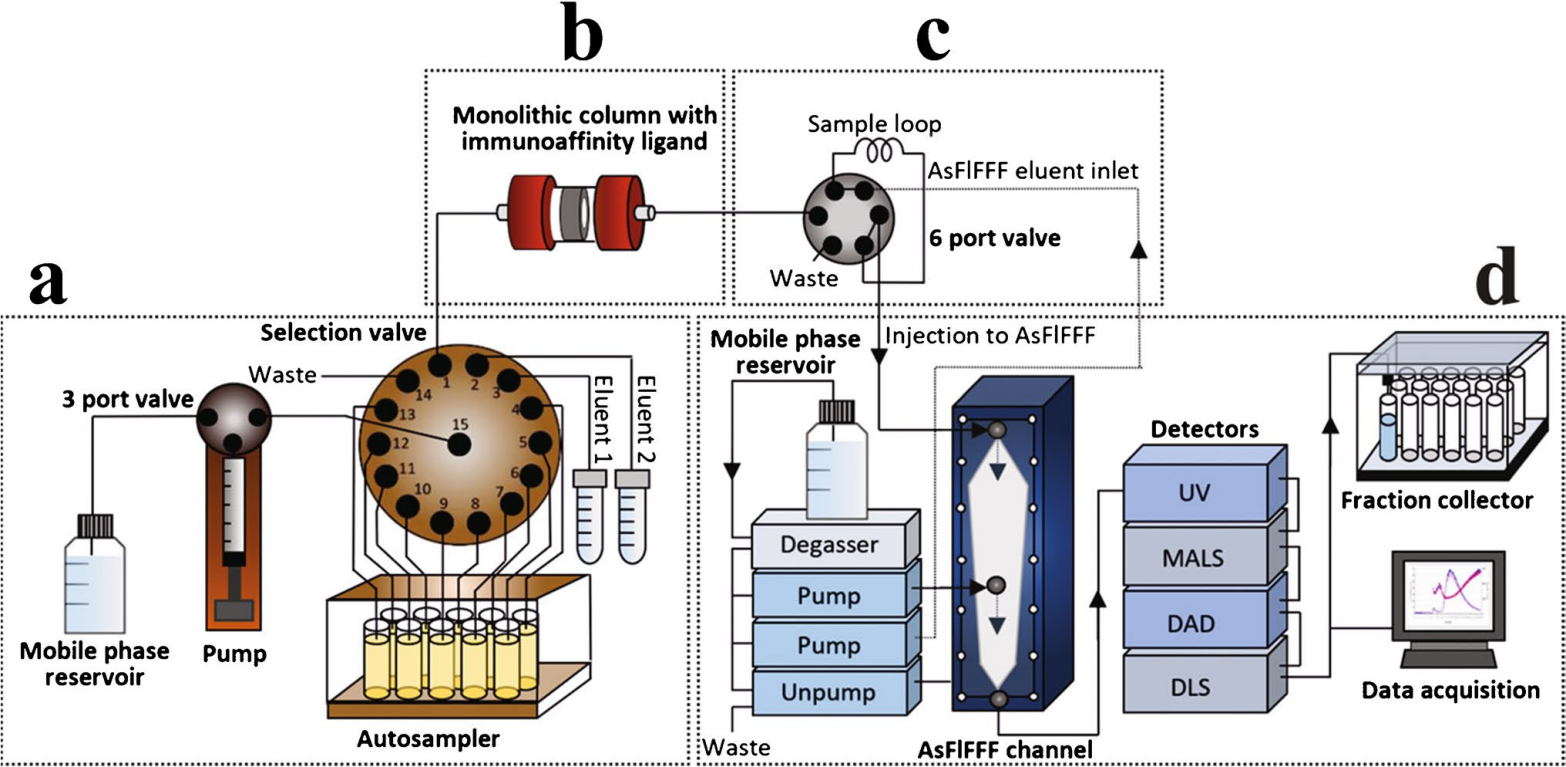
Schematic of an on-line coupled immunoaffinity chromatography-asymmetric-flow field-flow fractionation system for analysis of nano-sized biomolecules from plasma. The system is comprised of **a** a monolithic column for immunoaffinity chromatography, **b** an automated six port valve for injection to AsFlFFF, **c** AsFlFFF with ultraviolet, multiangle light-scattering, dynamic light-scattering, and diode array detectors, and** d** a fraction collector. Figure reprinted from [28]

Multia et al. [28] developed an on-line immunoaffinity chromatography coupled with an asymmetric-flow field-flow fractionation device, where anti-CD61 and anti-CD9 were used to selectively fractionate platelet-derived EVs from those originating from multivesicular bodies. As shown in Fig. 14, this involved a sample preparation section, immunoaffinity column, the AsFlFFF itself and multiplexed detection and analysis. The microfluidic channel separated three EV size ranges: < 50 nm, 50–80 nm and 80–120 nm [28]. For 5 mL of sample, the immunoaffinity stage took 51 min and the AsFlFFF subpopulation took 40 min.

#### Deterministic lateral displacement

**Fig. 15:**
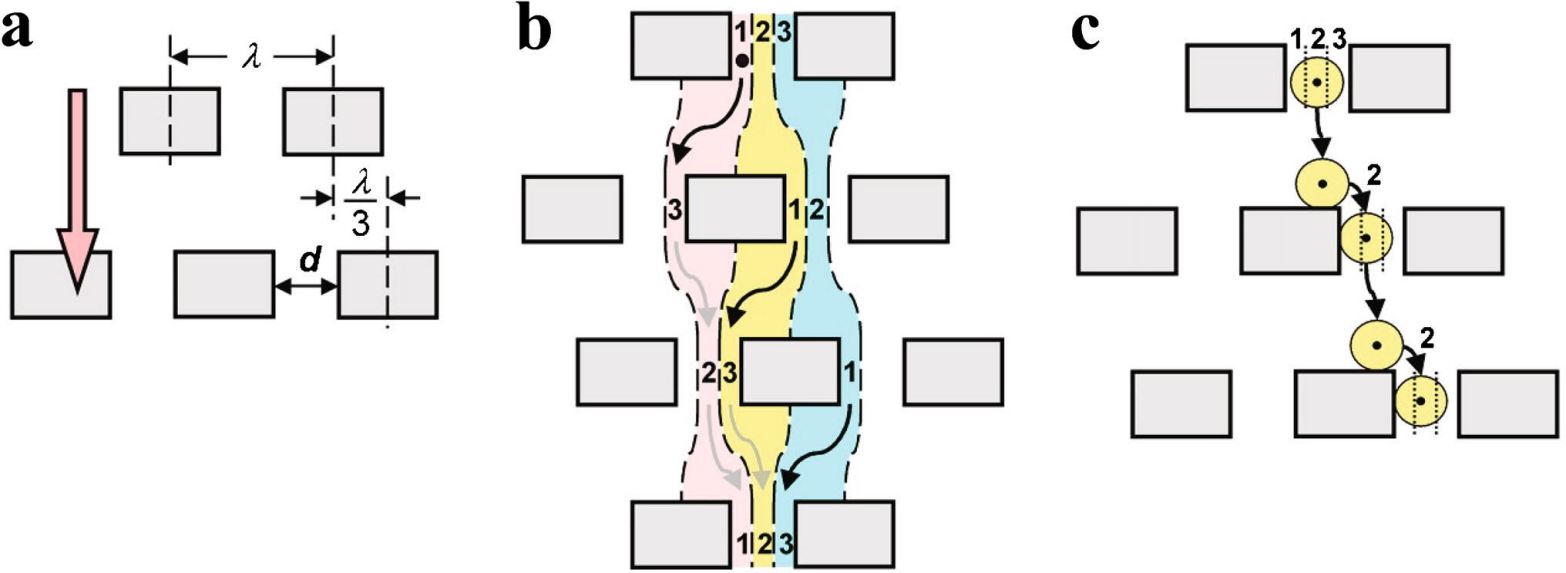
**a** Micrometre-scale obstacle array, where each row is shifted horizontally by $\frac{\lambda }{2}$, fluid flow direction shown in orange. **b** Fluid emerging from the gap in three parallel streams (red, yellow and blue). The first row’s lane 1 becomes lane 3 in the second row, which then becomes lane 2 in the third row of obstacles. **c** A particle follows the streamline, staying in the same lane (indicated by the black dot) and so is physically displaced at each row of obstacles. Illustration from [29]. Reprinted with permission from AAAS

Deterministic lateral displacement (DLD) is a microfluidic technique utilising laminar flow and bifurcations through a periodic array of obstacles, to enable predictable migration of particles. Huang et al. [29] designed a silicon chip which was able to spatially separate sub-micron particles (see Fig. 15). Particles smaller than the critical radius follow their stream path throughout the DLD array, whereas particles are displaced to the neighbouring stream by an obstacle if they are larger than the width of the stream path.

**Fig. 16:**
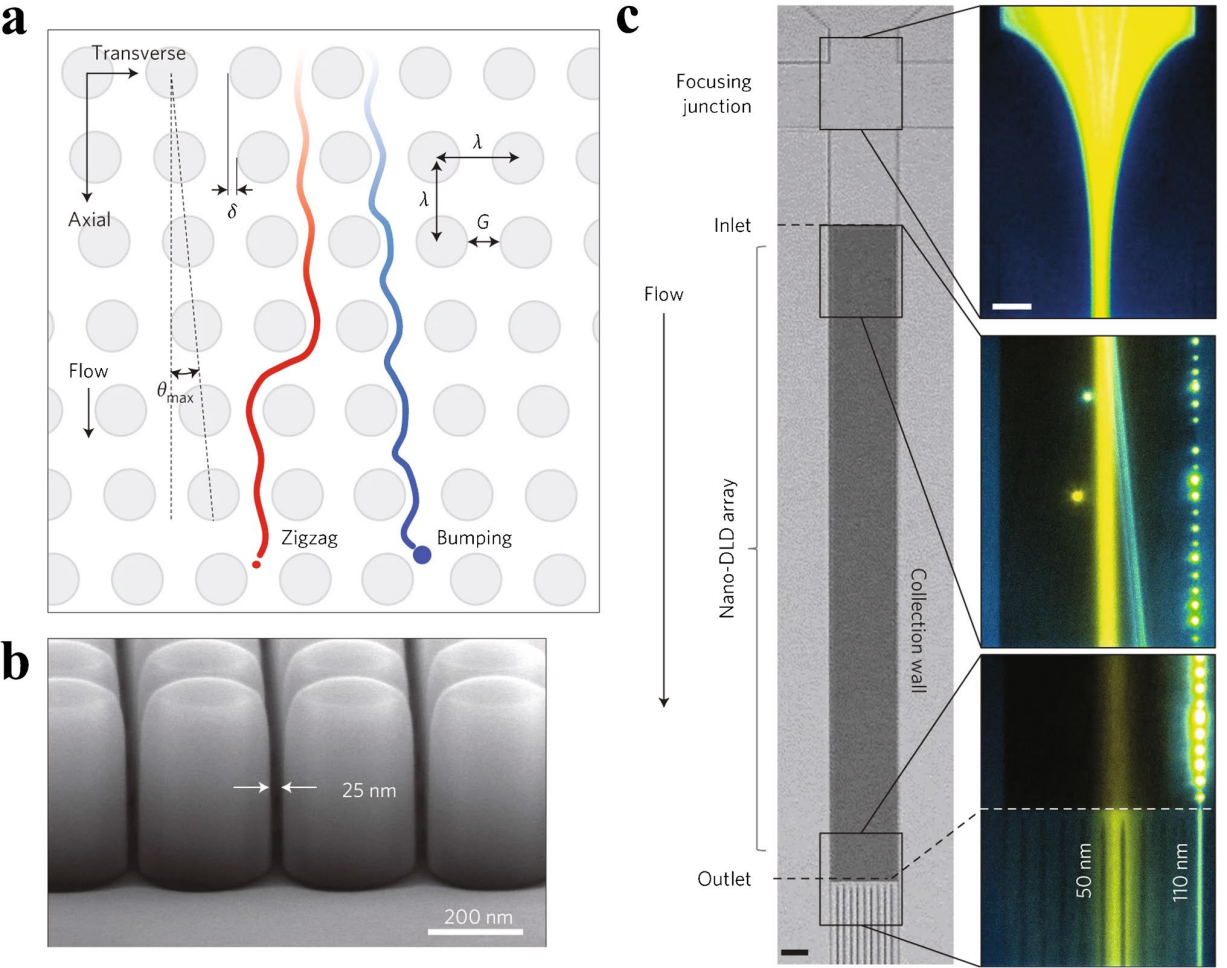
**a** Schematic of a DLD pillar array where particle trajectories follow a laminar flow in either zigzag mode (red) or bumping mode (blue). Only particles above a critical diameter will be displaced at the maximum angle by the bumping mode. **b** Scanning electron microscope image of the sorting array with separation 25 nm and row-to-row shift of 400 nm. **c** Microscopy images showing the device sections and continuous separation of 50-nm (yellow) and 110-nm (blue) beads, where the larger beads have been displaced to the right. Reprinted by permission from [30], Springer Nature, *Nature Nanotechnology*, Copyright 2016

The nano-DLD developed by Wunsch et al. [30], illustrated in Fig. 16, is an array of 25–235-nm separated pillars allowing separation of 110 nm from 20-nm colloids. When operated under continuous flow of 0.1–0.2 nL/min, human-urine-derived exosomes were separated into > 100-nm EVs in a bumped fraction and 20–100-nm EVs from the zigzag and partially bumped fractions. The sample took 30 min to reach the array, and the sample recovery was < 10 μL of small EVs and < 1 μL of large EVs. Smith et al. scaled up this technique in a massively parallel nano-DLD device with a total of 1.44 billion pillars per chip [31]. This enabled significantly higher throughput, and the device was able to isolate EVs from serum and urine at a flow rate of 15 μL/min, at a driving pressure of 1 MPa.

DLD has the advantage of being continuous flow and label-free; however, there are many practical disadvantages with this technique when used for nano-scale particle separation. DLD typically requires high operating pressure and gives modest throughput. Fabricating these devices is particularly challenging and costly, increasingly so as throughput is increased. Additionally, clogging can be an issue with these devices.

#### Viscoelastic separation

Viscoelastic separation devices are passive and utilise non-Newtonian fluids as described by Yuan et al. [32]. The principle is that the shear-thinning polymer-containing media induces an elastic force on nano-sized particles, pushing them towards the centre. A size-dependant equilibrium point is reached, and larger particles move closer to the wall due to the shear-gradient-induced lift force [33]. An early application of this technique used a cylindrical channel of non-Newtonian fluid to create a Lagrangian trap where the elastic forces migrate the nanoparticles laterally despite the opposition of Brownian motion and drag forces [33]. Similarly, albeit using sheath flow, Lui et al. [34] were able to separate large extracellular vesicles from exosomes, as the elastic force scaled to the second order with the particle diameter. Alternatively, a co-flow creating a Newtonian and viscoelastic interface utilised competition between interfacial elastic lift force and inertial lift force to drive selective migration from a biological sample into laminated viscoelastic fluid [35].

**Fig. 17:**
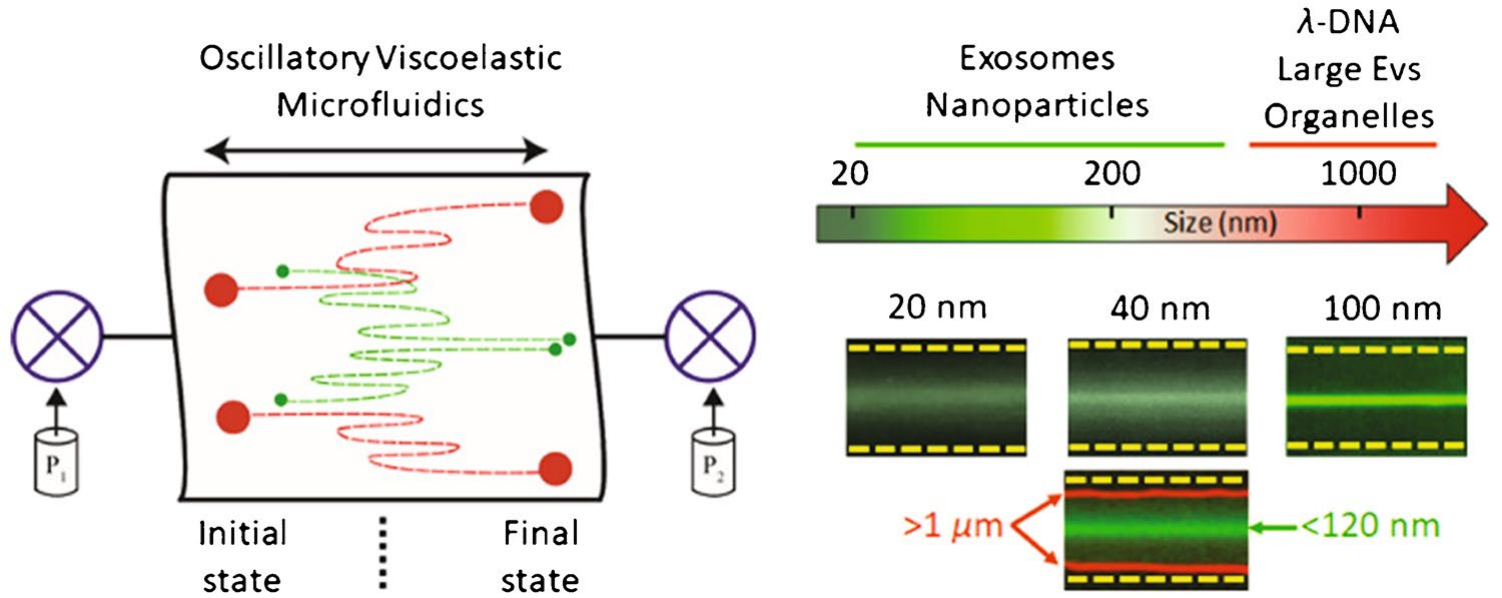
Schematic of the oscillatory viscoelastic separation principle. Pressures P_1_ and P_2_ oscillate the viscoelastic microfluidic system and result in size-dependent migration of particles. Milk fat globules > 1 μm (red) were pushed towards the walls, and small EVs < 120 nm (green) were focused towards the centreline. Reprinted with permission from [36]. Copyright 2020 American Chemical Society

A novel oscillatory viscoelastic device enhanced these devices by superimposing an oscillatory flow on a unidirectional flow, allowing equilibrium positions to settle in a shorter channel [36]. This device was only 4 mm long but was able to focus EVs in 20 s by rapidly oscillating the flow, thereby increasing the effective travel distance. Small EVs (122 nm mean diameter) were isolated from a mixture containing milk fat globules (1–2 μm) (Fig. 17) with 67% efficiency using oscillation frequency 2 Hz and 4 bar. Although this shows great promise, the fluid properties are critical to device performance so more research may be required before this can be used to isolate EVs directly from biofluids.

### Active devices

Active microfluidic devices involve external forces such as magnetic, electric or ultrasonic fields to drive separation with a low risk of clogging. Devices still incorporate hydrodynamic forces, inertial forces and diffusion; however, innovative techniques can allow particles to be separated by more than just hydrodynamic size and immunoaffinity.

#### Acoustophoresis

Acoustophoresis can be used to manipulate particles in a microfluidic system through primary radiation forces and scattered sound interactions. Acoustophoresis is a gentle way of isolating, enriching and washing particles and vesicles without labelling, which makes it suitable for biological samples. Devices utilising acoustic waves use either bulk acoustic wave (BAW) or surface acoustic wave (SAW) actuation. In BAW devices, a transducer vibrates the whole microchip at the channel’s resonant frequency, giving rise to a standing wave. In SAW devices, interdigital transducers (IDTs) generate acoustic waves that travel along a surface and couple into the fluid-filled channel.

SAW devices tend to operate at higher frequencies than BAW devices; this causes to a stronger radiation force able to isolate EVs in continuous flow. However, BAW devices have intrinsically higher acoustic energy densities which typically offers higher throughput.

**Fig. 18:**
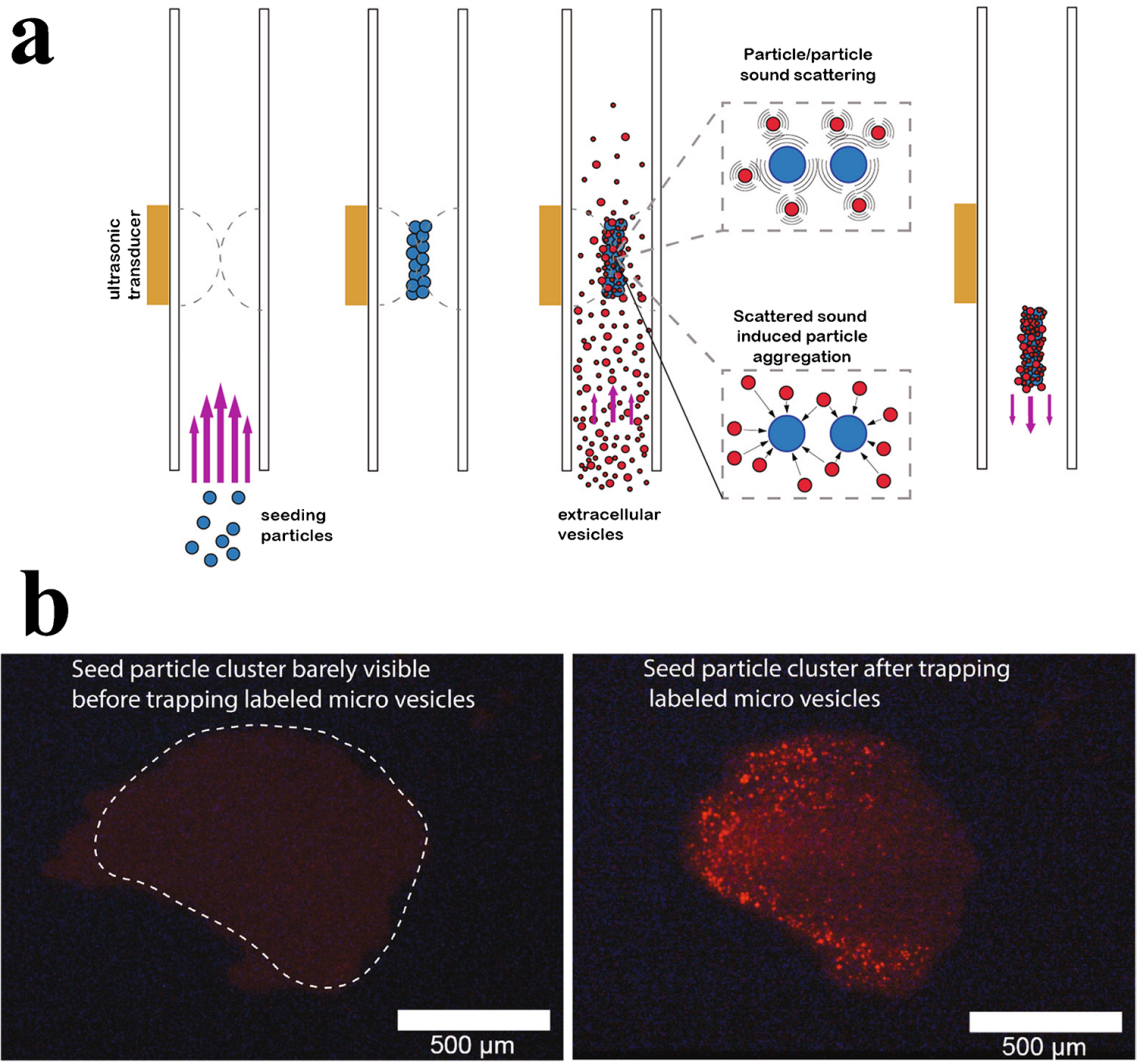
**a** Procedure for acoustic seed particle trapping of EVs. Seed particles are loaded in the acoustic field. EVs are caught in the seed particle cluster through scattered sound interactions. Following a wash, the particle cluster can be eluted by turning off the sound. Adapted with permission from [39]. Copyright 2018 American Chemical Society. **b** Images of particle trapping in an acoustic trap. Fluorescently (red) labelled vesicles are enriched in the seed particle cluster. Adapted with permission from [41]. Copyright 2016 American Chemical Society

**Fig. 19:**
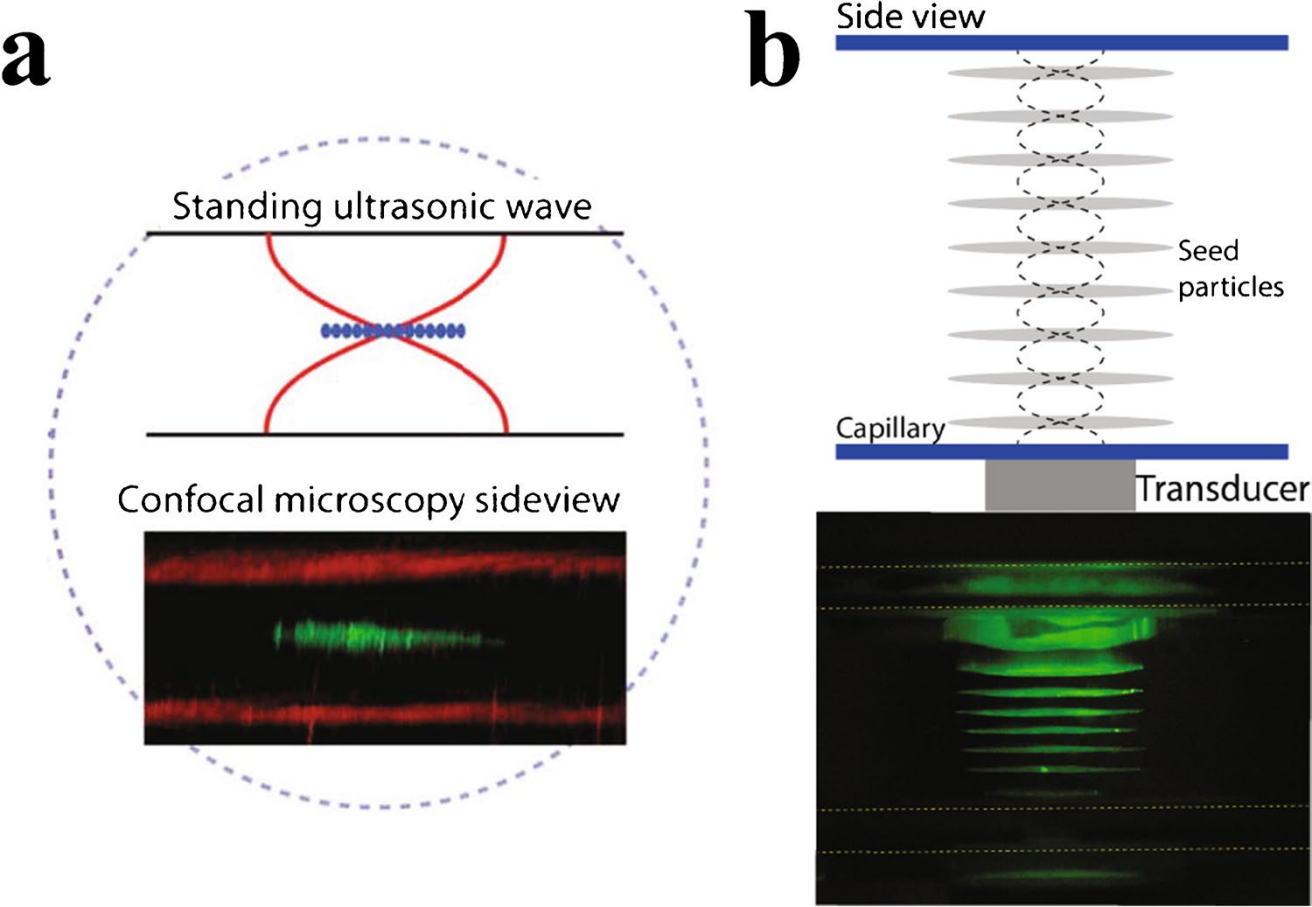
**a** Schematic and image of particle trapping in a single-node acoustic trap. Fluorescently (green) labelled particles are retained in a single cluster in the centre of the channel. Adapted and reproduced from [37] with permission from the Royal Society of Chemistry. **b** Schematic and image of particle trapping in a multi-node acoustic trap. Fluorescently (green) labelled particles are retained in multiple distinct clusters along the height direction of the channel, corresponding to the pressure nodes in the standing wave. Figure adapted and reprinted from [43]

Acoustic trapping is a technique for retaining particles in an acoustic field. In acoustic trapping with BAW actuation, a strong localised standing wave in a channel results in a stationary pressure node, where the primary radiation force can retain microparticles against flow. It is possible to trap nanoparticles by utilising scattered sound interactions from larger, preloaded seed particles [37]; see Fig. 18. The AcouTrap instrument (AcouSort AB, Lund, Sweden) has used this method to trap, enrich and wash EV from blood plasma [38–41], conditioned media [39] and urine [39, 42]. Acoustic traps will trap all particles that have a positive contrast factor, that is, more dense and less compressible than the surrounding medium. Once particles are acoustically trapped, it is easy to perform washes and buffer exchanges. A typical acoustic trap generates a single pressure node in the centre of the channel, Fig. 19A, trapping particles against a flow rate of 10–30 μL/min. A larger acoustic trapping channel has recently been developed to generate multiple pressure nodes, Fig. 19B, which enabled enrichment and washing of EVs at 500 μL/min. Several millilitres of urine was processed and yielded sufficient protein for subsequent EV proteome profiling using mass spectrometry [43].

**Fig. 20:**
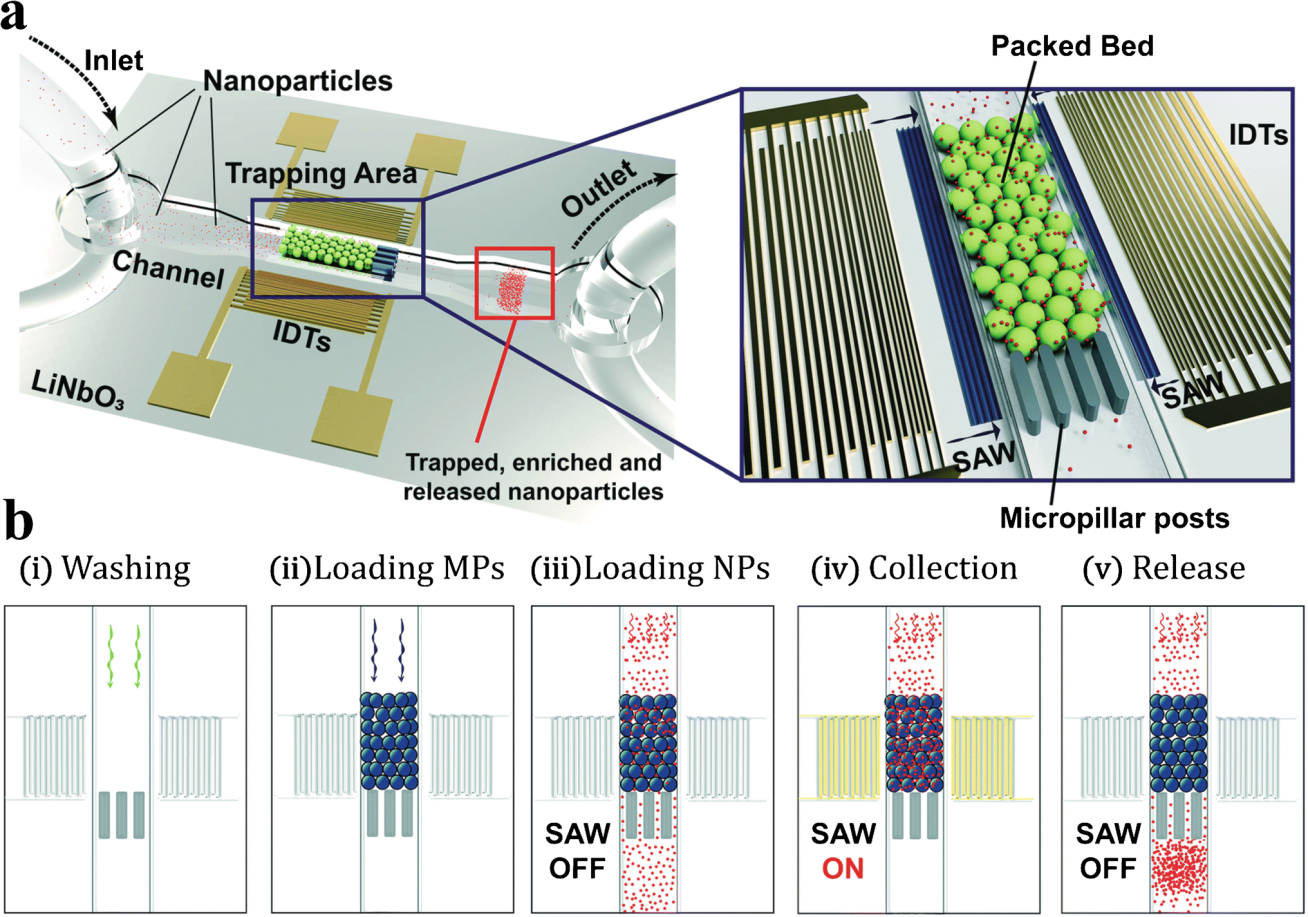
**a** Sound wave–activated nano-sieve illustration and **b** the corresponding process for trapping and release. Particles in a packed bed are actuated with surface acoustic waves at the particle resonance frequency. Scattered sound interactions allow for nanoparticle enrichment in the packed bed. The nanoparticles can be released by turning off the SAW. Reproduced from [44] with permission from the Royal Society of Chemistry

Acoustic trapping can also be performed with SAW actuation. A packed bed of seed particles confined with micropillar posts can be actuated by two opposing IDTs at the resonance frequency of the seed particles [44]. The scattered sound interaction between the seed particles and the nanoparticles causes the nanoparticles to aggregate in the packed bed, retaining them against flow; see Fig. 20. Habibi et al. [45] utilised these sound wave–activated nano-sieves (SWANS) to trap small EVs from cell culture supernatant at a flow rate of 0.1 μL/min.

**Fig. 21:**
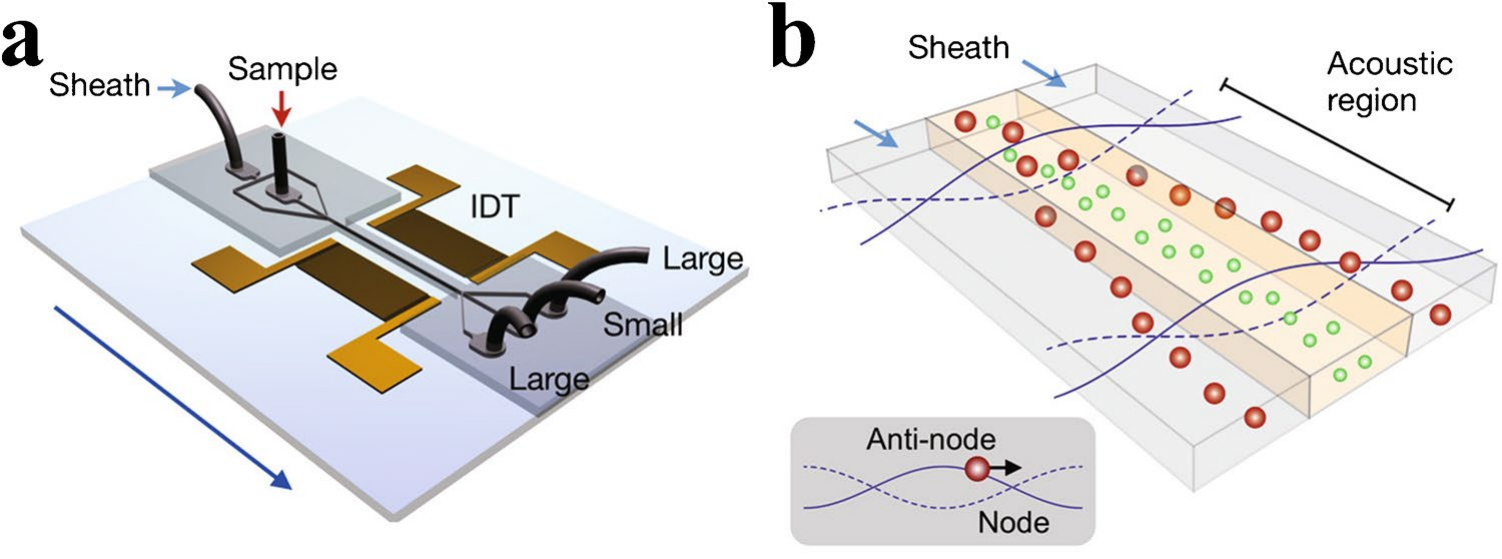
**a** SSAW device for EV separation. Two opposing IDTs generate a standing wave inside a fluidic channel. **b** Particles move towards the pressure nodes at different rates, depending on their size, allowing for separation of differently sized EVs. Reprinted with permission from [46]. Copyright 2015 American Chemical Society

Standing surface acoustic waves (SSAW) use opposing interdigitated transducers (IDTs) that generate a standing wave inside a channel. Microparticles move towards the pressure nodes at different speeds depending on their size, such that they can be deflected to different flow lines. Lee et al. [46] used this technique, under a continuous flow of 1 μL/min, to remove larger particles from a mixture, leaving a fraction of smaller particles in the original flow path, Fig. 21.

**Fig. 22:**
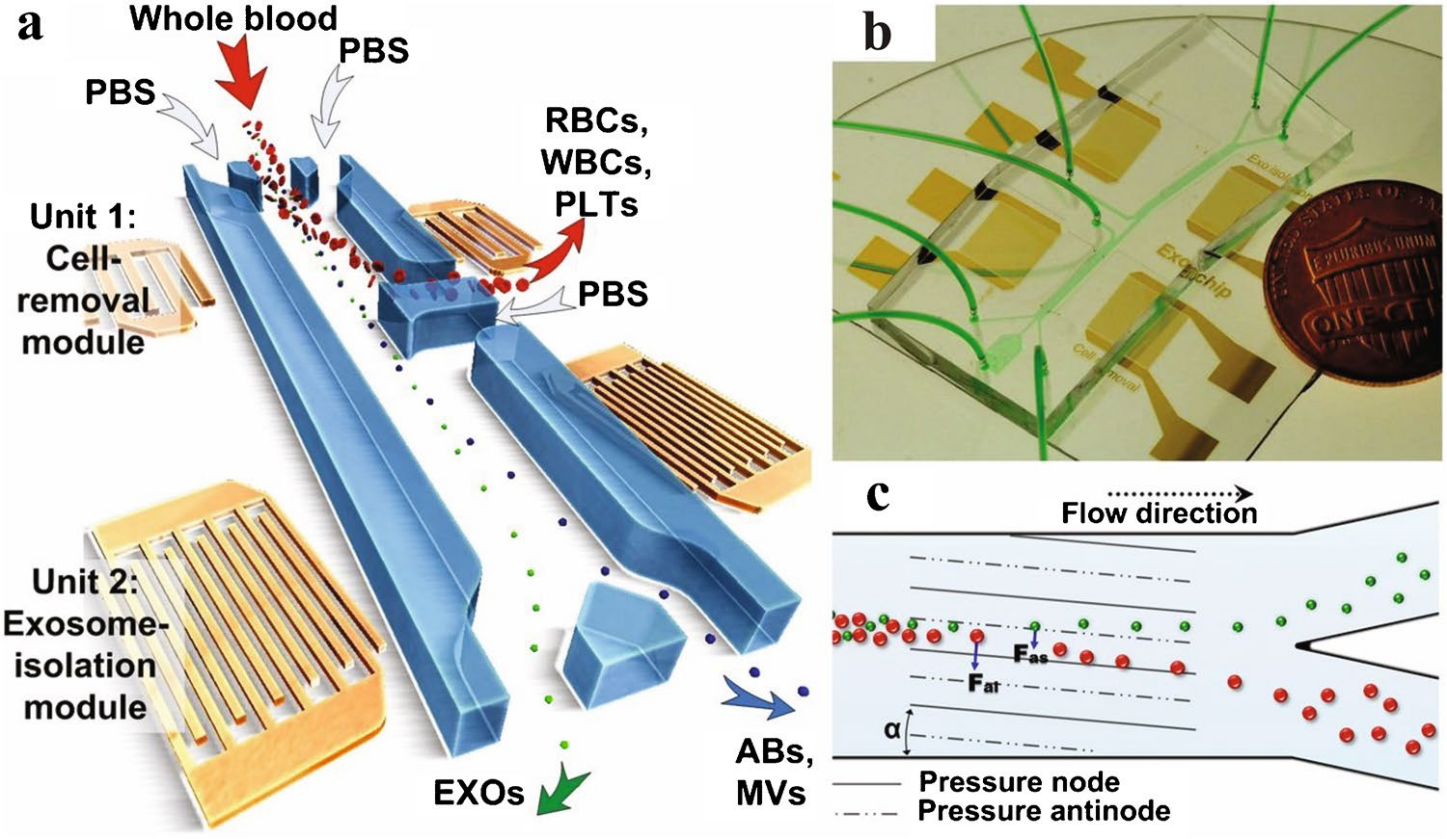
taSSAW device for separation of EVs and blood components. Particles will follow the tilted angle of the standing wave, deflecting them into other streamlines and allowing separation based on size. **a** Schematic of the device. Larger cell components are removed from whole blood in a first separation step, leaving EVs in plasma. **b** Image of the device. **c** In a second separation step, large EVs are separated from smaller EVs, generating one fraction containing small EVs and one containing large EVs. Figure reprinted from [47]

The tilted angle standing surface acoustic wave (taSSAW) is similar to SSAW, but its two opposing IDTs are tilted in relation to the fluidic channel. This results in angled pressure nodal planes in the channel which is used to deflect larger particles (experiencing a sufficiently large acoustic radiation force) from their streamlines as the Stokes’ drag force pushes them along the nodal planes. Wu et al. [47] developed a two-step purification taSSAW chip, shown in Fig. 22, through which large and small EVs could be separated from whole blood at a flow rate of 4 μL/min. However, the small EV fraction still contained plasma components and both fractions were significantly diluted by the sheath flow.

Whilst acoustic trapping enables washing, in SSAW and taSSAW, smaller vesicles will still be in their initial buffer (plasma or cell culture media) without removing background proteins. SSAW techniques sort particles by deflecting away the larger particles with radiation forces which do not affect small vesicles directly and are therefore not suited for enriching smaller EVs for protein analysis.

#### Magnetophoresis

**Fig. 23:**
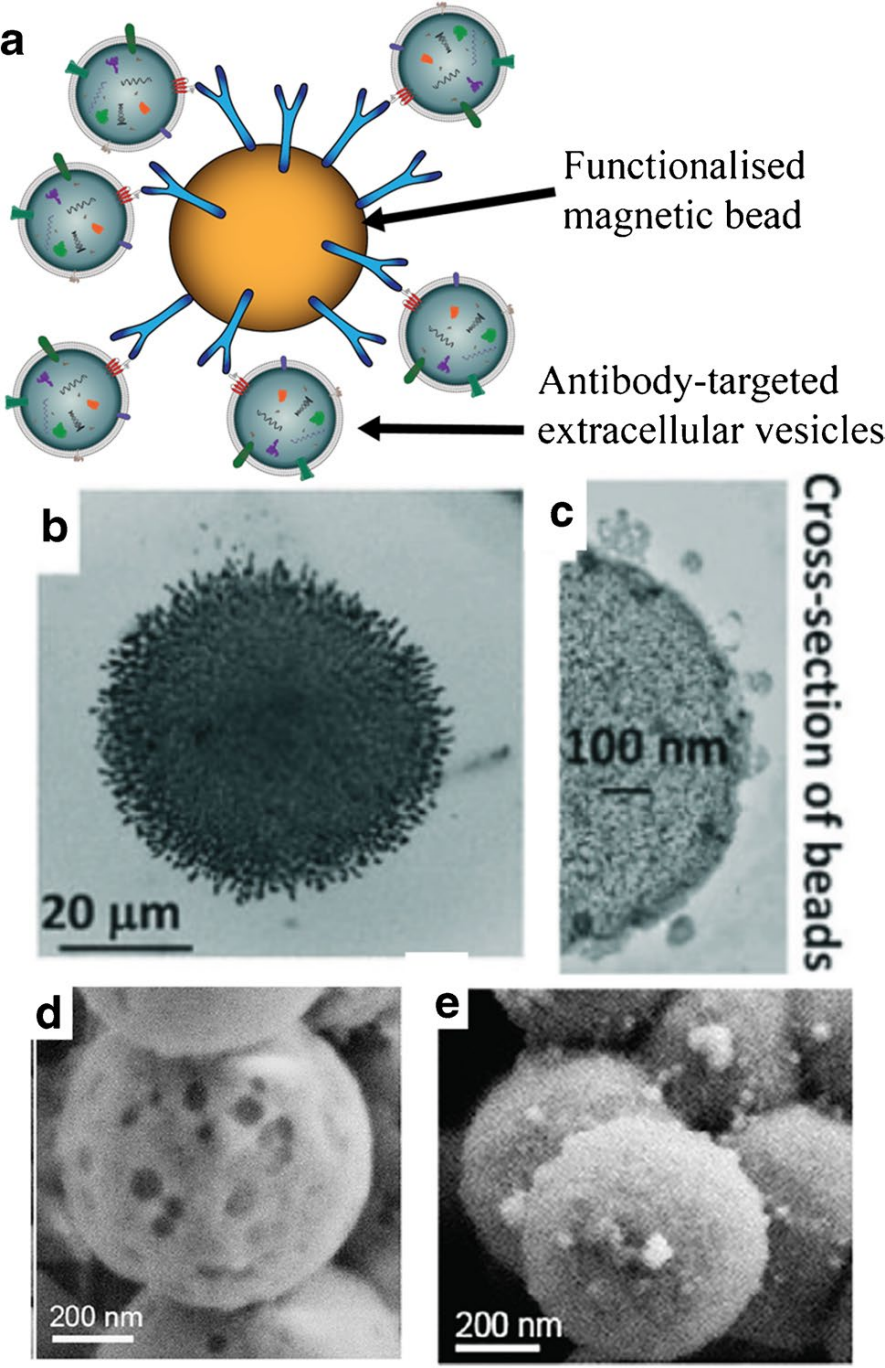
**a** Schematic of EV isolation with an immunomagnetic bead. **b**, **c** Images of an immunomagnetic bead with bound EVs and a cross-sectional TEM image of it. Images reprinted from [13]. **d**, **e** SEM images of a surface-imprinted magnetic nanoparticle before and after EV capture. Adapted with permission from [48]. Copyright 2021 American Chemical Society

Most magnetophoresis techniques used to isolate EVs exploit IC via magnetic beads, as shown in Fig. 23. Magnetic fields can enrich the beads surrounded by captured EVs more easily than with functionalised surfaces. Nanoparticles can search the sample volume very efficiently and have a large functional surface area which speeds up incubation time.

The ExoSearch chip was developed to facilitate the isolation of EVs with immunomagnetic beads from as little as 20 μL of plasma in 40 min [13]. The device was better than ultracentrifugation at isolating small EVs (80% compared to 61% of EVs were below 150 nm). The continuous flow mixing with slow flow rates (1 μL/min) means that isolated EVs are more likely to be intact than with UC [13].

In 2016, MagCapture Exosome Isolation Kit PS was commercialised. This kit utilised Tim4 activated magnetic beads that bind to phosphatidylserine displayed on the EV surface [49]. More recently, Fe_3_O_4_–EDC-NHS-NPs:anti-CD9 was found to be particularly stable and sensitive for EV isolation performed on a microfluidic device which could handle 500 μL of whole blood at a flow rate of 50 μL/min [50]. Yang et al. have developed artificial magnetic colloid antibodies which are not immune-based but utilise a surface-imprinting method to form a recognition layer onto a magnetic nanoparticle [48]. These highly specific beads allowed 90% EV capture efficiency after 20 min of incubation (Fig. 23d, e).

**Fig. 24:**
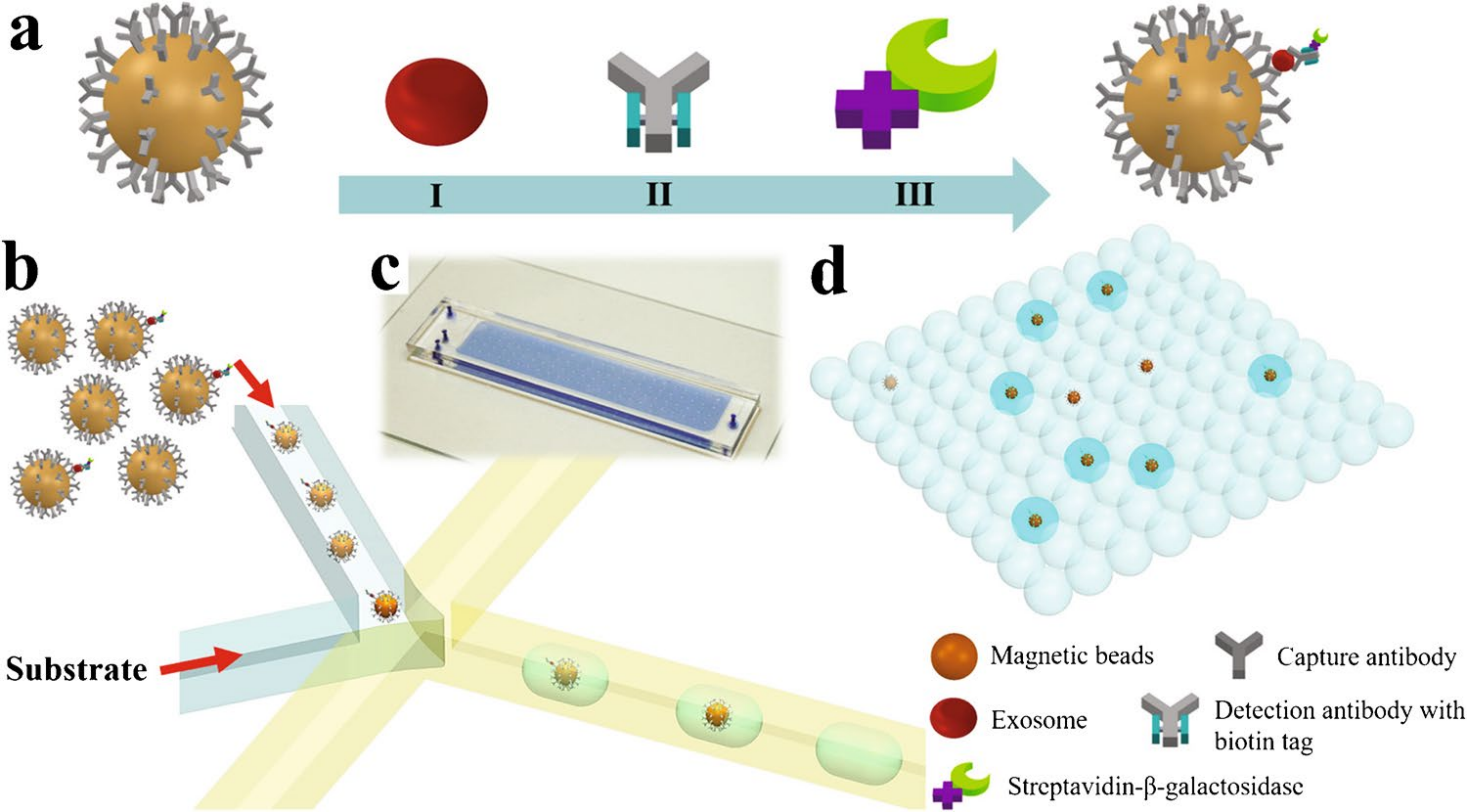
Schematic of droplet digital ExoELISA. **a** Magnetic bead bound to an EV via an immunocomplex, **b** microdroplet co-encapsulation, **c** microfluidic chip and **d** fluorescent readout. Reprinted with permission from [51]. Copyright 2018 American Chemical Society

More sophisticated microfluidic systems with integrated measurement include fluorescent [51] or digital [52] readouts. Lui et al. presented the digital droplet ExoELISA system (see Fig. 24) which confined a single exosome per droplet with a fluorescent enzymatic reporter and could detect 5–40,000 exosomes/μL [51]. Jeong et al. developed an integrated magnetic-electrochemical exosome platform (iMEX) where the magnetic beads in 8 channels were enriched at the electrodes with a detection limit of 3 × 10^4^ EVs from 10 μL of sample (compared with a standard ELISA protocol ~ 10^7^ EVs) [52].

A major limitation of these magnetic bead devices is that they require optimisation of the beads, functionalisation and incubation time. As with other immunoaffinity-capture-based techniques, this is limited by the performance of the antibody labelling and can only isolate EVs with those targets.

**Fig. 25:**
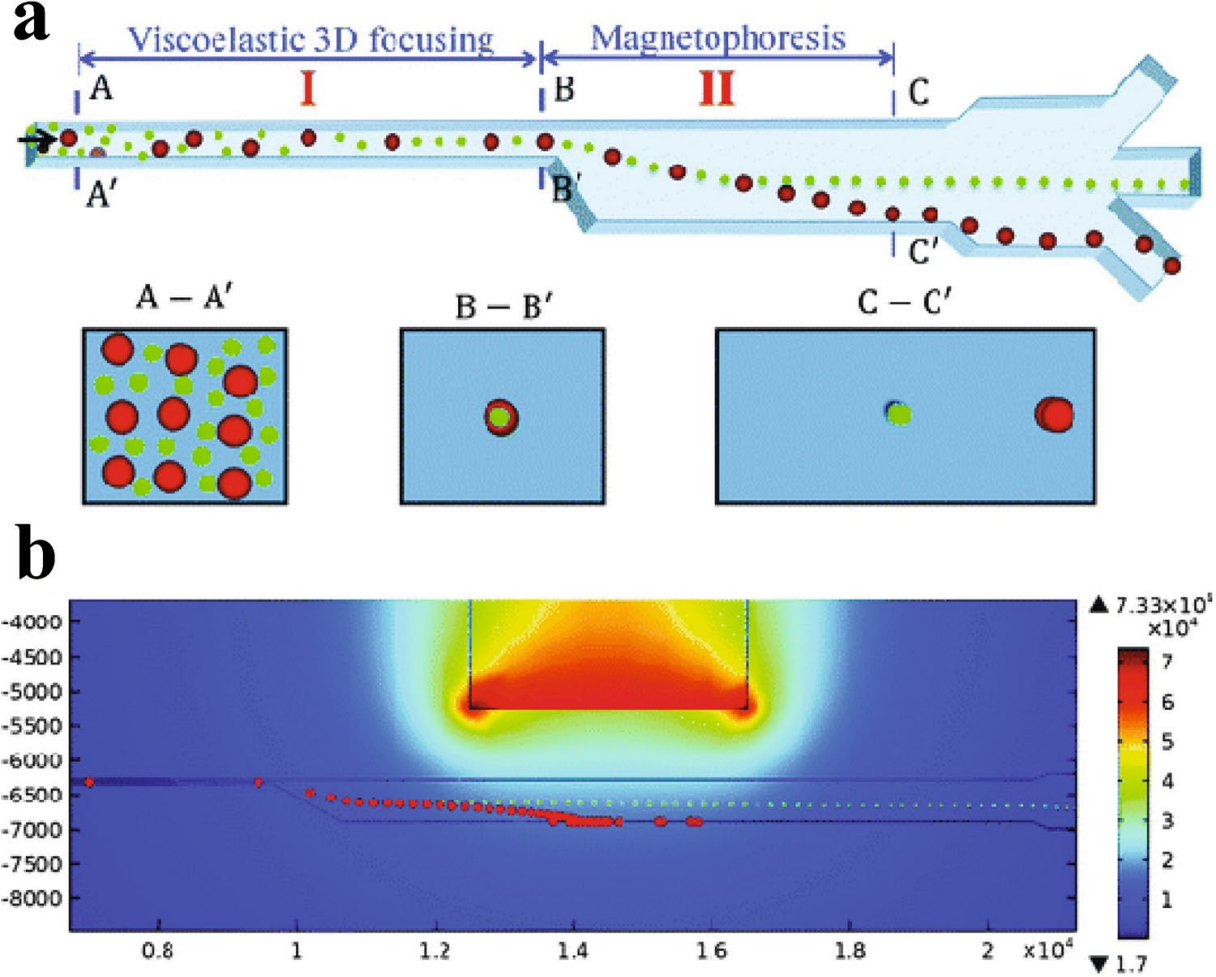
**a** Schematic of a microfluidic channel allowing separation of small (green) and large (red) non-magnetic particles by magnetophoretic force. **b** Corresponding numerical modelling of the particle separation in a non-uniform magnetic field generated by a permanent magnet. Reproduced from [54] with permission from the Royal Society of Chemistry

An alternative magnetophoresis method, ferrohydrodynamic separation (see Fig. 25), does not require EVs to bind to beads. By inducing a magnetic flux density gradient in a viscoelastic ferrofluid, diamagnetic particles experience a ferrohydrodynamic force proportional to their volume. Lui et al. used their FerroChip to separate EVs by size with a throughput of 1–3 μL/min [53]. Smaller EVs (~ 200 nm) were separated from larger EVs (~ 1000 nm) with 94.3% and 87.9% purity respectively.

#### Dielectrophoresis

**Fig. 26:**
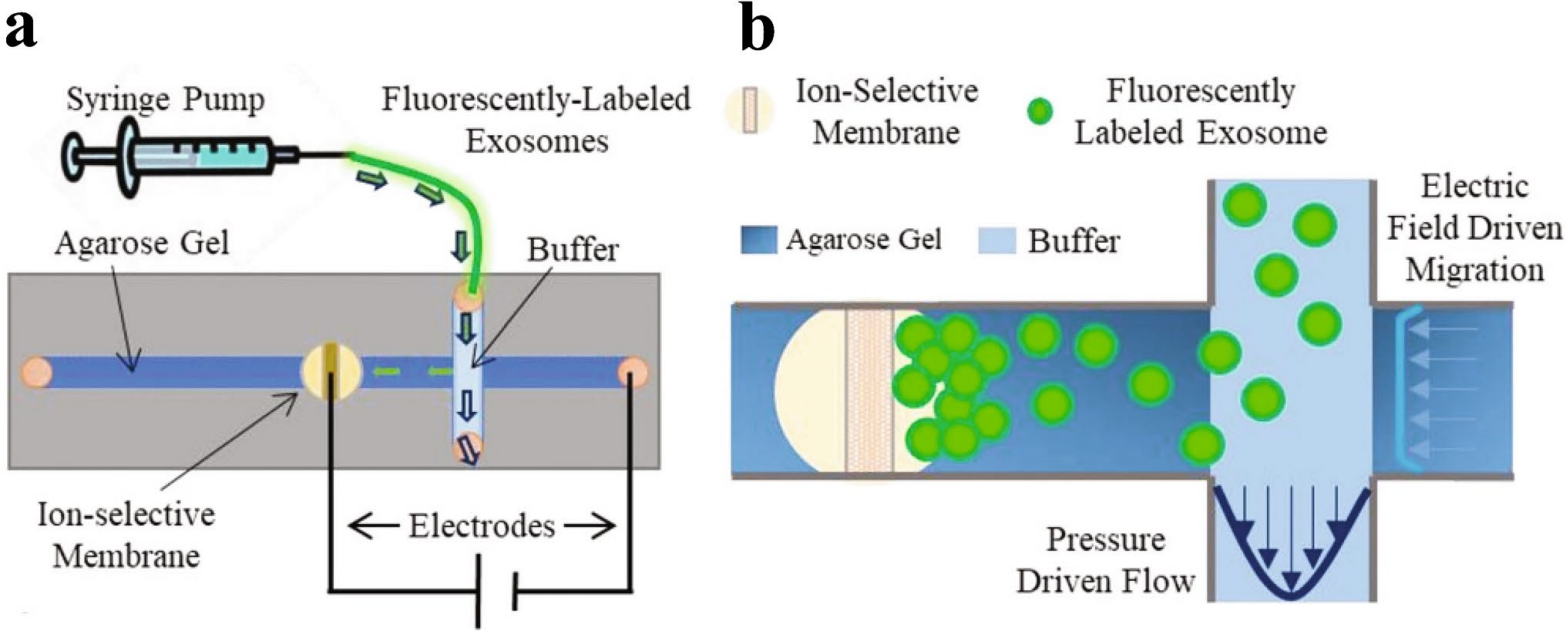
Dielectrophoretic isolation and enrichment of exosomes. **a** A solution containing purified exosomes is run through a microfluidic DEP device. **b** Exosomes are isolated in a gel with a pore size of 220 nm, stopping large particles from migrating into the gel region. The exosomes cannot pass the ion-selective membrane and are therefore enriched on one side of the membrane. Reprinted with permission from [57]. Copyright 2018 John Wiley and Sons

In addition to acoustic and magnetic forces, EVs can also be manipulated and isolated using electric fields. One such common technique is dielectrophoresis (DEP), wherein dielectric particles are polarised by an electric field gradient and then migrate either to the field minimum or maximum depending on the dielectric constants of the particle and the medium [55]. Chen et al. [56] demonstrated separation of exosomes from breast cancer cells and breast milk based on differences in membrane capacitance. Marczak et al. employed an electric field to direct EVs through a porous gel towards a cation-selective membrane, where they were enriched [57]; see Fig. 26. The device reportedly allowed approximately 70% isolation efficiency of small EVs at a flow rate of 3 μL/min. Larger EVs had lower isolation efficiency, perhaps due to the sieving effect of the gel or their lower mobility in the electric field [57].

**Fig. 27:**
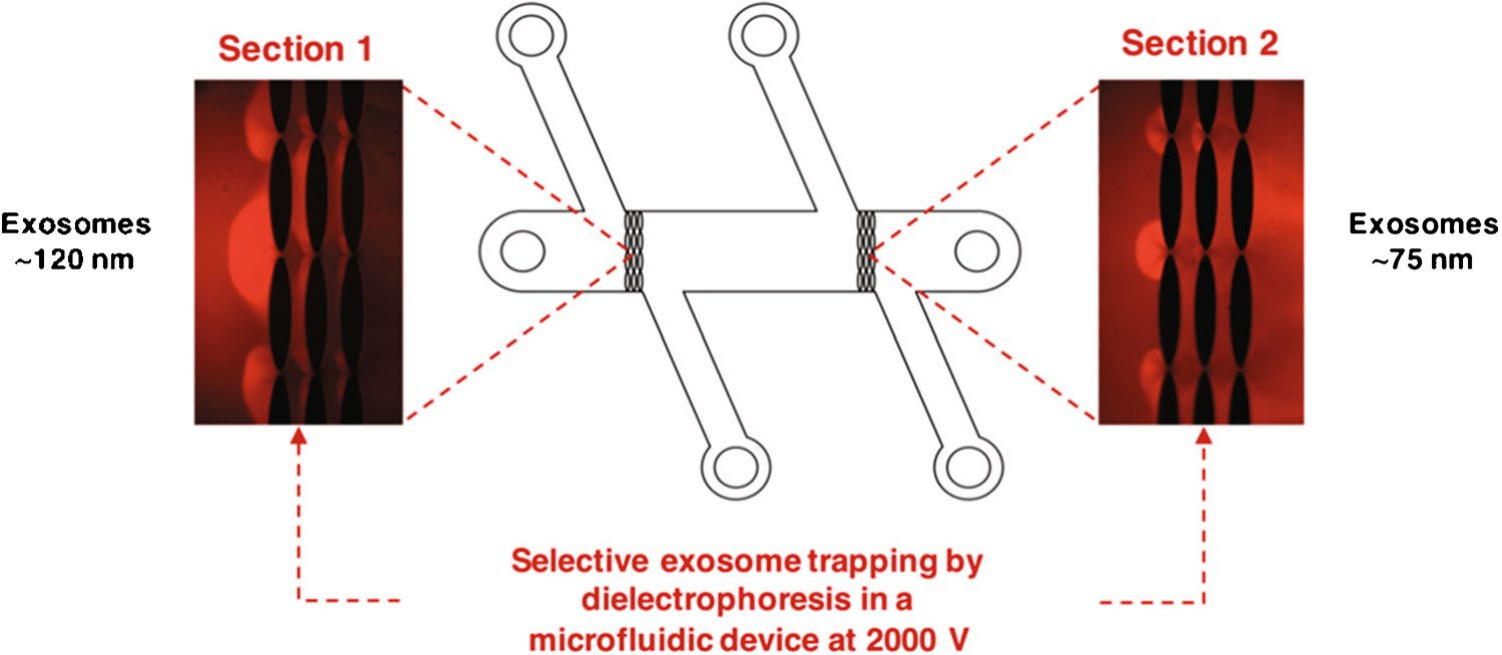
Size-dependent isolation of exosomes using dielectrophoresis with arrays of insulating posts. Reprinted with permission from [58]. Copyright 2019 American Chemical Society

Insulator-based dielectrophoresis (iDEP) introduces arrays of insulator posts between which the high electric field gradient can retain particles. Figure 27 shows how the spacing between posts has been used to separate purified exosomes into two population diameters of 113 ± 10 nm and 73 ± 9 nm [58]. One advantage of this system is that it is driven by electroosmotic flow, so it does not require syringe pumps. However, this device is unsuitable for samples containing cell debris because the small gaps may get blocked.

## Conclusions

Compared to conventional methods, microfluidic techniques in general allow for processing of smaller sample volumes. Additionally, they can allow for higher recovery and isolation of specific subtypes of EVs with fewer non-vesicular co-isolates, reducing background interference in subsequent biomarker analysis steps. Microfluidic devices vary widely in performance of EV isolation, as summarised in Table 1. Therefore, it is important to consider the requirements for downstream analysis and applications when choosing an isolation method. In addition to maximising throughput, recovery and purity, one must consider other factors such as capacity, sample volume, enrichment, co-isolates and potential for clogging. Furthermore, immunoaffinity-based techniques require expensive antibodies. Many microfluidic techniques require complex chip fabrication, yet if standardised, they could offer better reproducibility. Microfluidic isolation methods show great promise, with some commercial systems already available and improvements and new techniques being reported at a rapid pace.

**Table 1:** Performance summary of EV isolation techniques

Technique	Separation property	Throughput (μL/min)	Recovery	Purity	Sample type
Ultracentrifugation	Size, density	–	Med	Low	–
Precipitation	Size, surface markers	–	High	Low	Plasma and serum [7], urine [8]
SEC	Size	–	Med	Med	–
Mechanical filtering	Size	25–1000	Low–high	Med–high	Urine [18, 19], cell culture medium [17, 21], plasma and lung broncholalveolar lavage [17]
Functionalised surfaces	Surface marker	0.05–14	Med	High	Cell culture medium [10, 24], urine [22], serum [11, 12, 23], plasma [10, 14, 25]
Hydrodynamic focusing	Size	4.5–23	Med	Med	Plasma [28], cell culture medium [26, 27]
DLD	Size	< 15	Med	Low	Purified urine exosomes [30], serum [31], urine [31]
Viscoelastic separation	Size	3	Med	Low	Purified cell culture media EVs [34, 36]
Acoustophoresis	Size, density, compressibility	0.1–500	Low–high	Med	Whole blood [47], plasma [38–41], urine [39, 42, 43], cell culture medium [38, 39, 45, 46]
Magnetophoresis	Surface marker	0.8–3	Med–high	High	Whole blood [50], plasma [48], serum [13], cell culture medium [59], urine [59]
Dielectrophoresis	Size, charge	3	Med–high	Med	Purified EVs [57, 58]

Several patient cohorts have been included in studies with microfluidics-based isolation of EVs from cancer patients such as those with ovarian [13, 48, 60], prostate [61], breast [62], pancreatic [63], lung [22], bladder [18, 19] and human papillomavirus–associated oropharyngeal [64] cancers. Clinically characterised patient cohorts of 40–220 patients have also been studied with a focus on cardiovascular disease [65–67]. However, diagnostic failures [59] are still an issue within this developing field.

## Abbreviations

AsFlFFF: Asymmetric-flow field-flow fractionation
BAW: Bulk acoustic wave
DC: Direct current
DEP: Dielectrophoresis
DGF: Density gradient floatation
DLD: Deterministic lateral displacement
ELISA: Enzyme-linked immunosorbent assay
EV: Extracellular vesicle
FFF: Field-flow fractionation
IC: Immunoaffinity capture
IDT: Interdigitated transducer
NTA: Nanoparticle tracking analysis
PDMS: Polydimethylsiloxane
PEG: Polyethylene glycol
PEO: Polyethylene oxide
SAW: Surface acoustic wave
SEC: Size-exclusion chromatography
SEM: Scanning electron microscopy
SSAW: Standing surface acoustic wave
taSSAW: Tilted angle standing surface acoustic wave
TEM: Transmission electron microscopy
UC: Ultracentrifugation
